# With super SDMs (machine learning, open access big data, and the cloud) towards more holistic global squirrel hotspots and coldspots

**DOI:** 10.1038/s41598-024-55173-8

**Published:** 2024-03-03

**Authors:** Moriz Steiner, F. Huettmann, N. Bryans, B. Barker

**Affiliations:** 1 IUCN Small Mammal Specialist Group (SMSG), IUCN, Rue Mauverney 28, 1196 Gland, Switzerland; 2 IUCN Species Survival Commission (SSC), IUCN, Rue Mauverney 28, 1196 Gland, Switzerland; 3 EWHALE Lab-Biology and Wildlife Department, Institute of Arctic Biology, University of Alaska Fairbanks (UAF), Fairbanks, AK USA; 4 Oracle for Research, 2300 Oracle Wy, Austin, TX 78741 USA

**Keywords:** BIG DATA, Squirrels, Maxent, Super species distribution models (SDMs), R, Oracle, Super computer, Cloud modeling, Climate-change ecology, Climate-change ecology, Ecological modelling

## Abstract

Species-habitat associations are correlative, can be quantified, and used for powerful inference. Nowadays, Species Distribution Models (SDMs) play a big role, e.g. using Machine Learning and AI algorithms, but their best-available technical opportunities remain still not used for their potential e.g. in the policy sector. Here we present Super SDMs that invoke ML, OA Big Data, and the Cloud with a workflow for the best-possible inference for the 300 + global squirrel species. Such global Big Data models are especially important for the many marginalized squirrel species and the high number of endangered and data-deficient species in the world, specifically in tropical regions. While our work shows common issues with SDMs and the maxent algorithm (‘Shallow Learning'), here we present a multi-species Big Data SDM template for subsequent ensemble models and generic progress to tackle global species hotspot and coldspot assessments for a more inclusive and holistic inference.

## Introduction

Many species and their habitat needs are either ignored, understudied, or poorly known; effective conservation is virtually impossible as the status of the world’s biodiversity crisis reflects^1,2^. Albeit popular and ubiquitous, the world’s squirrels are part of that group^3^.

For the global squirrels (300 + species)^3,4^, the habitat needs and ranges are widely unknown, not mutually agreed on, and data are not made publicly available in most publications^5^. The only publication apart from^3^ that truly focuses on the global hotspots of squirrels (or a large group of squirrels) is^6^, where they described them as “The tropics, particularly the forests of south and southeast Asia, are hotspots of squirrel diversity; however, this region generates the fewest scientific publications on squirrels.”

This approach of unwillingness to conduct transparent and repeatable science by not using fully open-access and publicly shared data can actually be observed throughout most mandated governance bodies across the policy scale (from municipality to state, federal and the U.N.), across private enterprises, and NGOs up to the Science academies (see for instance Snow Leopards in^7^; examples for squirrels found in^3^).

To overcome basic presence data gaps mandated, data repositories like GBIF.org can be used, and now, also get support with increasing citizen science efforts as one of the largest data blocks in such repositories within just a decade. However, a wider global survey, assessment, and synthesis for target species data has rarely been accomplished before, and instead, predictive models from data mining are to be used as surrogates^8^. Within that concept, Maxent is a popular and relatively accurate rapid-assessment algorithm in the ‘shallow learning’ group within the growing spectrum of wider Machine Learning (ML) and Artificial Intelligence (AI) (see^9^ for an overview and over 100 algorithms). Thus far, common issues of insufficient computational processing capacity significantly limited such global Big Data assessments to the use of tools available to “everyone” such as PCs and laptops. Few data and low-end computational platforms without much progress create a spiral down with insufficient progress while better solutions have existed for over a decade but remain widely underused. To overcome these common and decade-old limitations, here we utilize cloud accelerated methods and show a workflow for progress.

Thus far, either such large computation capacities (supercomputers) were not readily available to the public, Big Data were not available, or software was not developed and used for them, and thus, one could not make use of their full potential^8^. Therefore, it is crucially important to share such Big Data methods, and underlying data sets in an open-access fashion for updates and to gradually overcome this bottleneck with as many global species as possible. In general, the greatest science can perhaps be performed, but it remains of lower impact without sharing the data and results transparently for assessment, transparency, and repeatability (Open Access)^10^.

Another constraint is the habitat data necessary to actually run SDMs; the use of more than 20 habitat predictors in a good pixel resolution, well aligned and with a geographic projection, remains rare to tackle real ecology questions. Digital habitat data for species like squirrels are even less widely found and shared^5^. Similarly to^11^, here we compiled and used the best publicly-available 132 GIS layers set from various sources (see complete dataset in Chapter 3^3^).

By working on a cloud hardware, in this study, we present and assess a powerful but still somewhat simplistic workflow opening cloud computing applications further and allowing a sheer infinity of data to be processed with ‘shallow learning’ to set the stage for multi-species data mining and subsequent predictions and wider ML/AI ensemble models (e.g. see^12–15^). Here, we investigate the first global multi-species assessment with extraordinary novel amounts of data (“Big Data”), leading to in-time high-accuracy Super SDMs that were not possible to be created previously. Here, we focus on the global squirrel hotspots and coldspots as examples. As of now, there are no publicly-available hotspot/ coldspot maps available for all global squirrel species, explicit in space and time, especially not in a multi-species composite aggregate for the entire family, created with hundreds of thousands of occurrence points and 100 + environmental predictors, based on machine learning algorithms. Using such exhaustive digital tools and open-access data allows for global insights and sets the stage for a new global quantitative, repeatable, and testable standard, Super SDMs.

## Methods

We created a global SDM assessment of all the world’s squirrel species utilizing Machine Learning algorithms powered by Cloud Computing. This study builds upon a workflow and data previously introduced by^3^ and expands on that approach and workflow using almost three times as much new data. This workflow has been presented in Fig. 2 below. To our knowledge, this presents the highest number of predictors and occurrence records ever used for one SDM (see^14^ for 80 predictors, and^11^ for 100, and for multi-species models see^3^ for over 130). This moves Maxent from a simple ‘shallow-learning’ SDM algorithm into authentic data mining. We thus like to call it a Super SDM with the following method steps.

### Big data: occurrence data

**Figure 1:**
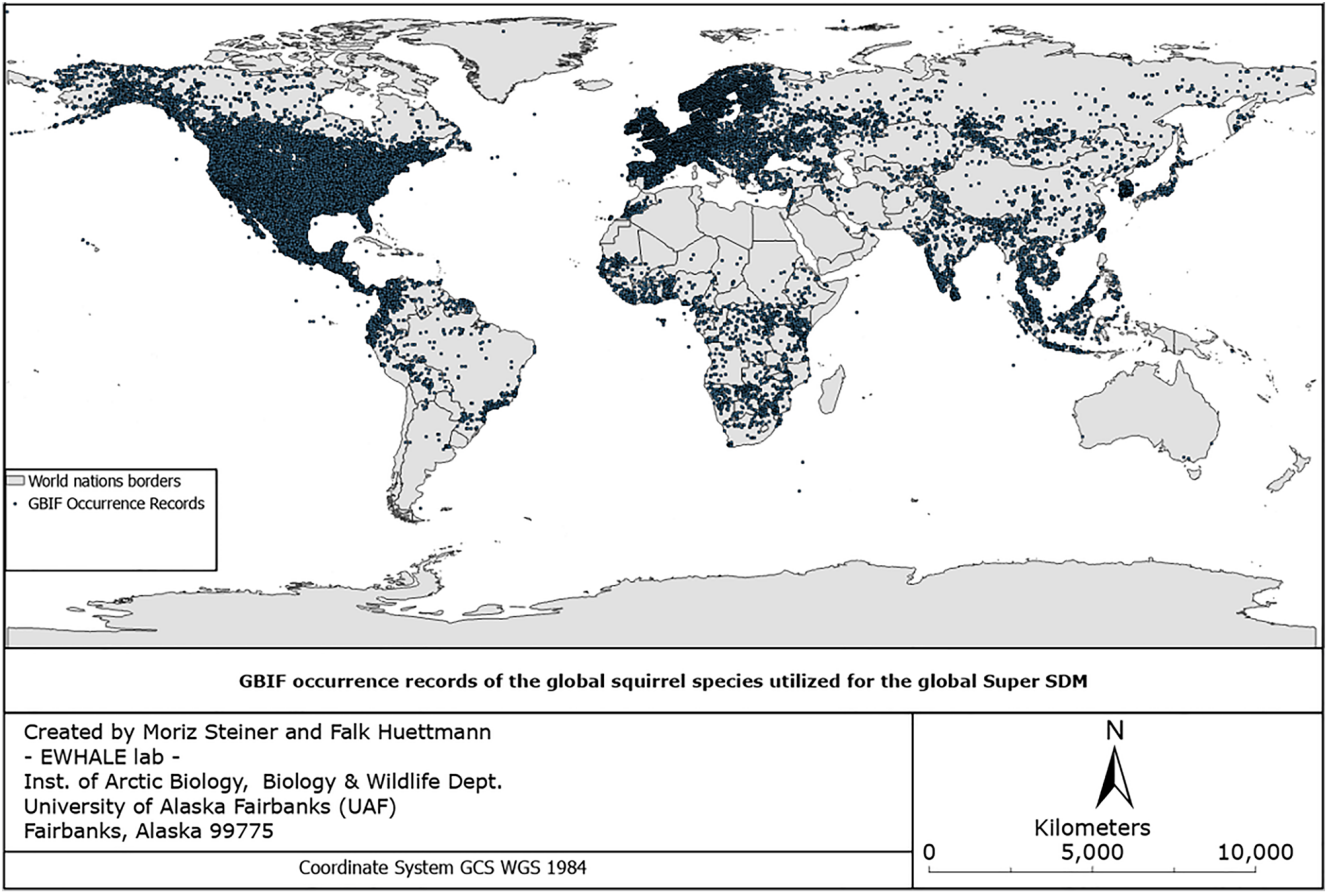
Occurrence points of all global squirrel species (300 +) utilized for the global squirrel SDM downloaded from www.GBIF.org.

We utilized all publicly-available online GBIF occurrences for the family Sciuridae (= squirrels) with a cut-off date of November 13th 2022 (www.GBIF.org receives constant new data submissions and updates its sets monthly). An older version of this downloaded dataset was used by^3^ in 2020 but was significantly updated and now contains a total of 1,543,980 raw occurrence points (see download 10.15468/dl.2banfj). These occurrence points have been obtained from GBIF utilizing the RGBIF package in R. The R script that has been utilized to obtain the occurrence points can be found in Appendix S1. After obtaining the occurrence data from RGBIF, we removed duplicates in the dataset in order to make it easier to handle the model run. There are different approaches to using ‘double locations’ as those are ‘true’ data^8^; however, Maxent is commonly known as a rather ‘shallow learning’ data mining tool which mostly relies on parsimonious concepts and creates by default its own pseudo-absences, also relying on a high number of background points^16,17^. Arguably, for our objectives, the duplicated occurrence points have assumably little influence on the global SDMs when all occurrences are combined, which we decided to do in order to create the global hotspot/ coldspot analysis for all squirrel species. After removing duplicates (utilizing “removing duplicates” function in MS Excel), we also removed all records without a geographic location and a described species name^18^, after which the dataset was saved as a CSV file and imported in the data directory to be accessible for the cloud hardware. This data preparation is necessary for Maxent's algorithm, which sets it apart from more advanced and deep-learning methods such as boosting (TreeNet) or bagging workflow etc., that are better able to work with raw and messy data within which the corresponding Machine Learning algorithm seeks patterns^19,20^. This resulted in 665,529 final occurrence points which have been mapped and presented in Fig. 1 below; see Appendix S2 for ISO-compliant metadata describing this unique resource. This final dataset does not contain the same amount of occurrence records for all species. Rather, it contains many more records for western and common species compared to non-western rare species. This is an artifact of the dataset and represents the current global reality and data availability of the sciences. It additionally highlights the data gaps of the world’s squirrels, which are to be filled and improved in the near future to create even more sophisticated and improved global models^3^.

Figure 1 shows the utilized occurrence points for this study, retrieved from GBIF.org. A detailed list of all included squirrel species and their corresponding record counts can be found in Appendix S3.

### Environmental predictors

Here, we utilized a total of 132 environmental predictors; a set that has been firstly partially compiled by^11^ and first presented as the world’s most complete socio-economic habitat predictor set by^3^. Here it has been re-utilized for this study. A detailed description of all predictors and their sources can be found in Appendix S4 (reproduced from Table 3.2 from^3^). A large number of predictors (in this case 132) aims to reflect the complexity of nature as inclusively and accurately as possible (‘holistic'). By only utilizing a handful or much fewer selected predictors, the number of untested hypotheses increases with every predictor left out. Biases increase, and interactions remain untested. Another reason for using so many predictors is that the predictor selection and their consecutive contribution to the models are supposed to be carried out by the machine learning algorithm rather than by the opinion of a human (parsimonious selection of predictors). A well-trained dataset, used by a robust Machine Learning algorithm with hundreds of interactions will most likely, in every case, find the most suitable predictors for the given dataset. It is a reason for the strength of Maxent and ML/AI. With fewer initial predictors, this process is biased to a larger extent, the number of testable hypotheses is smaller, and the models cannot fully benefit from the Machine Learning algorithm’s potential.

### Cloud modeling

In order to process the high quantities of data utilized for this study (point data and habitat layer data), we performed all modeling steps in a powerful Oracle Cloud Infrastructure computing instance (cloud.oracle.com) using the R environment for easy reproducibility.

Thanks to a computing grant to FH in 2022, we were able to use the ORACLE cloud; we used the settings depicted in Table 1.

**Table 1:** Oracle cloud settings utilized for global squirrel SDM.

Oracle cloud metric	Description
Computer system	Linux
Computer memory	1024 GB
OCPU count	64
Machine shape	VM.Standard.E4.Flex

Utilizing the settings presented in Table 1, we used Powershell on a local Windows laptop to remote access the cloud compute via SSH, and run an R script for the global Super SDM (see Appendix S5), virtually synchronized with the Oracle Cloud Infrastructure. This SDM has been created utilizing Maxent (version 3.4.4—https://biodiversityinformatics.amnh.org/open_source/maxent/) and the software packages “raster”, “dismo”, “rgeos”, “sp”, and “rJava” (see corresponding references in the sequence of the included packages^21–25^. In order to subsequently produce the desired SDM, we ran the commands “maxent” and “predict” in R via the SSH connection. To diminish possible data gaps as much as possible, we utilized 80% of the available data for training the ML model and the remaining 20% and 500 iterations for the model testing. This ratio of data attributed to training and testing is commonly found in literature but many models use a ratio of data for the model training that is smaller (sometimes significantly smaller) than the model testing ratio^26^. With our approach, we believe to have diminished possible data gaps as much as possible while still testing the model sufficiently with the remaining 20% of the data and 500 iterations.

**Figure 2:**
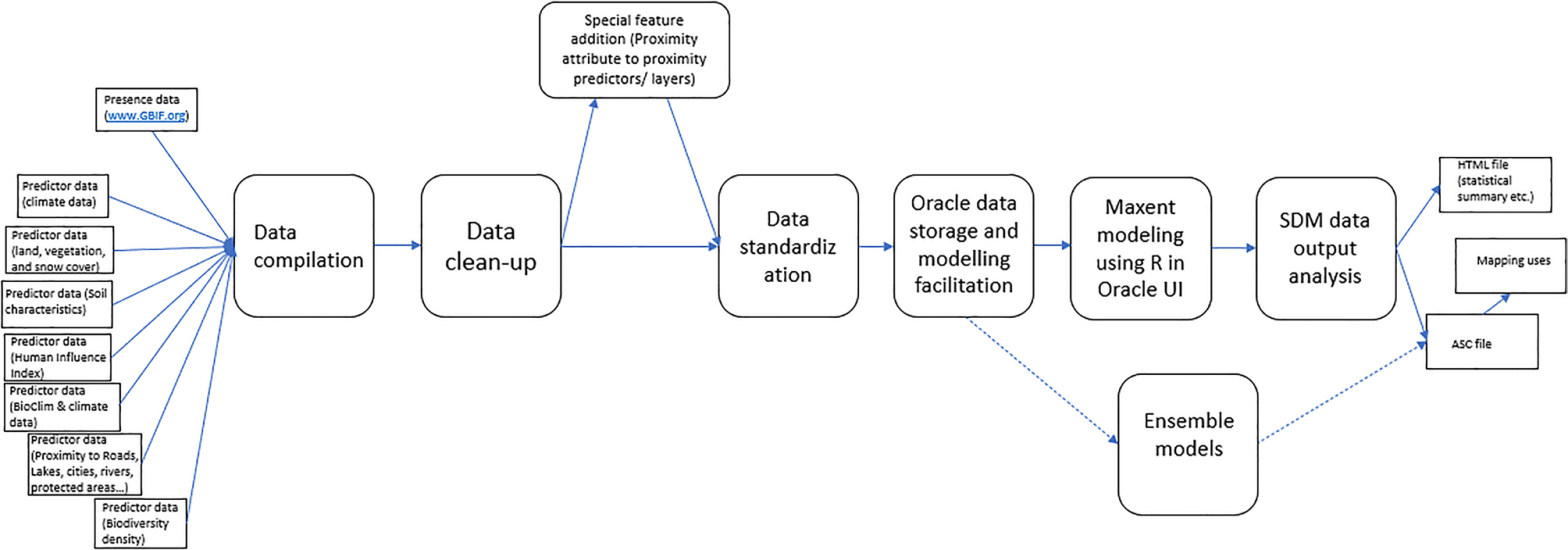
Methodological workflow global super SDM.

An overview of the workflow performed in this study is displayed in Fig. 2. This workflow includes all steps performed in the creation of the Super SDM in this study. It starts with the collection of the required datasets and ends with the results of the SDM in GIS. Additional add-on options are also included in this workflow, e.g. the option to create ensemble models. This workflow can act as a template for future Super SDMs studies, assessing other vertebrate species.

### Hotspot/coldspot identification

Once the SDM has been created, the produced raster has been imported into Open-Source GIS (QGIS version 3.10.6, obtainable via https://www.qgis.org/en/site/forusers/download.html); we also used ESRI ArcGIS for some operations. In GIS, with a visual rapid-assessment approach, we identified the global squirrel hotspots and coldspots. This distribution hotspot/ coldspot identification aims to show the predicted species distribution index of all global squirrel species (multi-species distribution index). Regions with a prediction index ≤ 0.32 have been classified as ‘coldspots’ (low prediction occurrence), and regions with a prediction index ≥ 0.66 have been classified as ‘hotspots’ (high prediction occurrence). These thresholds have been set up in this manner to represent the low 1/3^rd^ of the predicted occurrence index as coldspots with low predicted occurrence, a certain average or medium, and the top 1/3^rd^ of the predicted occurrence index as hotspots with high predicted occurrences. Because our work is fully open access, any of these settings can be re-visited and improved upon new data and research.

## Results

### Worldwide squirrel open access data compilation

We were able to compile and use the best-available point data in the world for 351 species included in the GBIF dataset. This set of methods is the first of its kind and allows many applications for SDMs and conservation management, all described with ISO-compliant metadata (see Appendix S2) allowing transparent and repeatable research.

### Oracle maxent run of a super SDM

With these extremely high numbers of utilized data (‘Big Data’), and the extraordinary computational power of cloud computing, without such a cloud modeling approach, this workflow would have not been possible to complete on a laptop or a PC—Windows 10 processor Intel® Core™ i5-4300U. But using cloud computing, we were able to achieve an output for this complex data cube after 7 full days of run time. After the prediction commands are finished, the produced global SDM has been exported into the data directory accessible for the cloud computer, from where the SDM raster has been downloaded. This produced raster (TIFF) file can be found in Appendix S6 and can be used in any OpenSource GIS application. According to our “evaluation” command and the Maxent results, we obtained the model diagnostics displayed in Table 2 below. These diagnostics describe the single-best result obtained by the standard procedure and default Maxent SDM algorithm^22^.

**Table 2:** Global squirrel Super SDM model evaluation.

Evaluation criteria	Description
AUC (area under the ROC curve)	0.9543
Correlation	0.4198
Test accuracy	0.6169

In addition to the model diagnostics, we also obtained the variable importance of the predictors as an outcome from the Maxent run. The top 25 predictors (judged by their model contribution) of our global Super SDM can be observed in Table 3. This can help to assess the relevance of GIS predictors and for specific data gaps, data improvements, and hypotheses tested in the field.

**Table 3:** Global squirrel Super SDM variable importance.

Variable	Percent contribution	Permutation importance
HII1	43.7	21.4
BIO19_2_5min	18.4	4.2
World_MAX_RH_JAN	13.5	0.2
WorldProtectedAreasMerged4	11.6	30.8
GlobalRoadsProxy2	4.1	10.7
Prec11	1.3	0.4
WorldSlope1	1.1	1.0
GlobalCities2	1.0	5.8
Prec09	1.0	2.5
srad8	0.8	1.2
srad4	0.8	3.6
tavg2	0.6	1
tmax12	0.6	1.4
WorldThreatenedMammalDensity3	0.5	8.2
BIO12_2_5min	0.2	0.2
World_MAX_RH_DEC	0.2	0.9
Prec08	0.1	2.5
srad10	0.1	1.0
FFJun2020_3	0.1	1.3
Prec01	0.1	0.1
GlobalRiversProxy2	0	0
WCaltitude	0	0
BIO3_2_5min	0	0.7
GlobalBigRivers11	0	0.4
srad7	0	0
BIO14_2_5min	0	0.1
srad11	0	0.5
World_MAX_RH_AUG	0	0
tavg4	0	0

Table 3 shows that the predictors ‘HII1’ (Human Influence Index), ‘BIO19_2_5min’ (Precipitation of Coldest Quarter), ‘World_MAX_RH_JAN’ (Global Maximum Relative Humidity for January 2020), and ‘WorldProtectedAreasMerged4’ (Proximity to the world’s protected areas) have been most contributing to our Super SDM. This indicates that the HII (Human Influence Index) predictor dominates the global squirrel hotspots and coldspots distribution. Apart from that, the most contributing predictors of the model can be classified as climate predictors. However, instead of focusing on these variable importance rankings, here we promote the approach from Leo Breiman, allowing inference from predictions^27^, asking to infer from the specific predicted pixel attributes. More work can be done on those pixels but here we make our prediction available and start this process.

Our map is the first for the 300 + squirrel species showing global hotspots and coldspots based on 132 predictors. It allows it to be more inclusive, complete, and holistic regarding the predicted outcome.

### Squirrel hot- and coldspots

**Figure 3:**
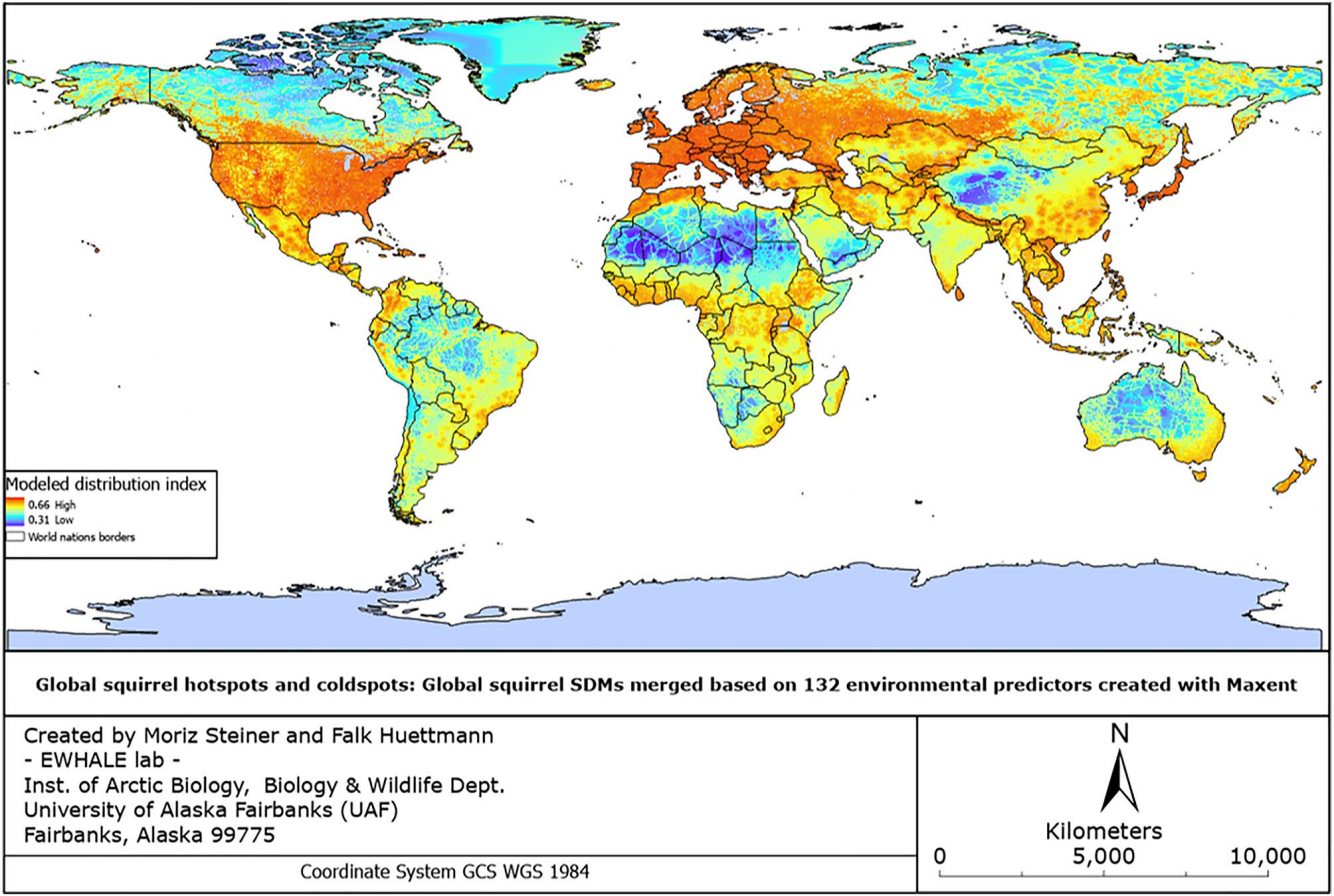
Global squirrel Super Species Distribution Model created with Machine Learning algorithms in the Oracle cloud computer.

The obtained Super SDM was then imported into ArcGIS Pro 3.1 (version 3.10—with a valid license downloadable via https://pro.arcgis.com/en/pro-app/latest/get-started/download-arcgis-pro.htm), where the symbology of the produced SDM was adjusted and map details were added. The resulting map is presented in Fig. 3.

In Fig. 3, we can observe that the major global squirrel hotspots are located in North America, Middle America, Europe, Southeast Asia, Japan, Northwestern Africa, whereas the global coldspots can be observed in the Sahara Desert (Africa), Tropical Region of South America, North American Arctic, Mongolia and Tibet, Southwestern Africa, Australia, Siberia, and the Middle East. Table 4 outlines all the identified global hotspots with a comment on the reasons for those regions to be considered hotspots, and Table 5 outlines the identified global coldspots with additional comments on the reasons for those regions to be considered coldspots. These tables represent the outcome of a literature review we performed to support our model results.

**Table 4:** Global squirrel hotspot regions.

Regions	Included countries	Reason(s) for high occurrences	References
North America	USA, Southern Canada	Originating grounds (= long evolution time), close to Anthropocene (parks, bird feeders, etc.), temperate and optimal climate for mammals, plenty of habitat diversity, prey abundance	^3,28–33^
Europe	Portugal, Spain, United Kingdom, Ireland, France, Belgium, Netherlands, Germany, Denmark, Switzerland, Liechtenstein, Luxembourg, Austria, Italy, Slovenia, Poland, Sweden, Norway, Finland, Slovakia, Czechia, Hungary, Croatia, Romania, Serbia, Moldova, Ukraine, Bosnia and Herzegovina, Albania, Montenegro, Bulgaria, North Macedonia, Greece, Latvia, Lithuania, Estonia, Belarus	Close to Anthropocene (parks, bird feeders, etc.), temperate and optimal climate for mammals, plenty of habitat diversity, prey abundance	^3,28,31,32,34^
Central America	Mexico, Guatemala, Belize, Honduras, El Salvador, Nicaragua, Costa Rica, Cuba, Haiti, Dominican Republic, Puerto Rico, several island states	Pristine tropical habitats, extraordinary habitat diversity, a high number of different possible ecological niches, and prey abundance	^35–38^
Northwestern Africa	Morocco, North Algeria, Tunisia	High human impact (benefits of living close to the Anthropocene), ideal for arid-loving species (predominately ground squirrels)	^39,40^
Western Asia	Georgia, Armenia, Azerbaijan, Iran, Pakistan, Afghanistan, Turkmenistan, Tajikistan, Kyrgyzstan, Kazakhstan, Western Russia, Northern India, Nepal, Bhutan	High habitat diversity with significant altitude changes. Hotspots are often observed close to areas with high human impact	^7,40,41^
Most eastern Asia	South Korea, Japan, Taiwan	Close to Anthropocene (parks, bird feeders, etc.), temperate and optimal climate for mammals, plenty of habitat diversity, prey abundance	^3,28,31,32,34^
Southeast Asia	Vietnam, Thailand, Laos, Cambodia, Sri Lanka, Indonesia, Brunei, Malaysia, Philippines	Pristine tropical habitats, extraordinary habitat diversity, a high number of different possible ecological niches, and prey abundance	^42–48^
Tropical Africa	Ethiopia, Western Kenya, Uganda, Rwanda, Burundi, Tanzania, Congo, DRC, Equatorial Guinea, Cameroon, South Sudan, Southwestern CAR, Nigeria, Benin, Togo, Burkina Faso, Ghana, Ivory Coast, Liberia, Sierra Leone, Guinea	Pristine tropical habitats, extraordinary habitat diversity, a high number of different possible ecological niches, and prey abundance	^49–52^

**Table 5:** Global squirrel coldspot regions.

Regions	Included countries	Reason(s) for low occurrences	References
North American Arctic	Alaska (USA), Canada	Unfavorable climate (too cold temperatures), low feed availability	^3,51^
Greenland	Greenland	Unfavorable climate (too cold temperatures), low feed availability	^3,51^
South America	Southern Venezuela, Guyana, Suriname, French Guinea, Southwestern Colombia, Peru, Northeastern Brazil, Bolivia, Northern Chile, Argentina	Few Squirrels have reached that far south throughout evolution	^3,53–55^
Southwestern Africa	Angola, Eswatini, Namibia	Unfavorable climate (too hot temperatures, and too arid)	^3,51,56^
Sahara and Sahel desert (Africa)	Central and Southern Algeria, Western Sahara, Mauritania, Northern Mali, Niger, Chad, Sudan, Libya, Southern Egypt	Unfavorable climate (too hot temperatures, and too arid), low feed availability	^3,51,56^
Middle East	Southern and Northern Saudi Arabia, Western Oman, Eastern Yemen	Unfavorable climate (too hot temperatures, and too arid)	^3,51,56^
Siberia and Tibet	Western China, Central and Eastern Russia	Unfavorable climate (too cold temperatures), low feed availability	^3,51^
New Guinea	Indonesia, Papua New Guinea	Squirrels did not reach these regions yet (see Wallace Line)	^3,57–59^
Australia and Oceania	Australia, New Zealand, Solomon Islands, New Caledonia, Fiji, Vanuatu, and several island states	Squirrels did not reach these regions yet (see Wallace Line)	^3,57–59^
Antarctica	Antarctica	Unfavorable climate (too cold temperatures), low feed availability	^3,51^

Despite the extraordinarily large numbers of occurrence points and environmental predictors, we still observe a certain degree of overprediction with the Maxent application in this study^60^. We can observe such overpredictions in e.g. Iceland or New Zealand (see Fig. 3 above). Arguably, this can indicate a vacant niche and squirrel species extinctions. It warrants further research.

## Discussion

We aimed to predict the latest state-of-the-art and high-accuracy distribution hotspots and coldspots of over 300 squirrel species using more than 130 environmental predictors in the form of a Super Species Distribution Model (‘Super SDM’). This Super SDM is based on a Machine Learning algorithm, applied to a Cloud Computing environment, aiming to improve the understanding of the world’s squirrels’ hotspots and coldspots with resulting science-based conservation progress.

Squirrels are marginalized. Tree-living squirrels are of conservation concern with ongoing old-growth and forest loss worldwide. Data are widely missing, specifically for tropical species, where most of the diversity sits. Here we were able to benefit from the citizen-science database GBIF.org. Further, we were able to use and expand on the Open-Access Data layers and the workflow introduced by^3^. Big Data exist but remain widely underutilized^61^. Further, in GIS and SDM models it is common to miss habitat layers; the relevant and needed set of habitat predictors remains incomplete while ML/AI can often overcome those gaps. Additionally, ML/AI methods are likely to perform best in capturing species-habitat associations as a large number of habitat-associated predictors are included in the models, allowing for inclusive and holistic predictions. While our work opens up new avenues, it is far from complete. However, as a new workflow, it presents a minimum estimate, we can exclude uncertainty for 132 predictor layers adding overall ‘certainty’ to the model predictions based on open-access Big Data, the Cloud, and Machine Learning.

The evaluation criteria of the Maxent multi-species composite model indicate a near-perfect model fit with an AUC (Area under the ROC Curve) of 0.9543. Besides this great result, the Correlation is 0.4198, and the Test accuracy is 0.6169, which would indicate a rather high Sensitivity and low Specificity. However, with the setup of this composite model, these results can be attributed to the large diversity in the input data set (e.g. many different species with varying occurrence records), and therefore do not pose any major over-prediction issues. Overall, here one aim was also to have a quantifiable outcome, provide the best-available data, and start a discussion on the global hotspots and coldspots of all squirrels (as a group/ composite) based on actual data, rather than creating the ultimate species-specific SDMs with the least overfitting possible (see for other models in comparison^3,62,63^. We are following a Macro-Ecology perspective to provide progress on the wider issues, globally.

This research and the workflow open up new avenues worldwide for SDMs, the use of SDMs, and the use of datasets that exist but are widely underused and under-analyzed. We actually think that not running Super SDMs is by now poor-inference science, e.g. when just relying on HSI, BioClim, Occupancy, or RSF models run on a PC or laptop, and it should become a baseline for any defendable habitat assessment and policy. With methods and data at hand now, it easily becomes best-professional practice and sets a mandate for more conclusive habitat models, as well as for SDMs for any species, e.g. for IUCN and industrial impact assessments, including climate change predictions. Arguably, SDMs with less than 100 predictors and few occurrence records in the public and open access realm despite decades of research are of inferior value and should be re-run with this compiled habitat data set made available by us (see an example for Tree Kangaroos in Papua New Guinea^64^, and forthcoming).

Here, a new world is attempted and envisioned where computing-intensive methods are a research requirement, parsimony is ended (see also^8^), and results are more inclusive and holistic allowing for improved inference (see^8,14,65^). With this workflow introduced here, decades-old SDM limitations can be overcome, and Big Data high-accuracy predictions can be created (see^66^ for 1m resolution). Here, for reproducibility, we utilized the most common, free of charge, and widely used SDM algorithm Maxent, which can be considered part of the shallow learning ML tools, but with the large amounts of data and the cloud computing efforts, with these methods, the SDM can still be considered a high-accuracy top-class SDM. Nonetheless, utilizing other software that can generally be considered as ‘deep learning’—if applied correctly—(e.g. TreeNet/ Random Forest), and Neural Networks, etc. (see^67^), that commonly do not require much data cleaned-up, would likely provide even more accurate results. Other network-based systems are also expected to have a critical impact on data processing and the implementation of AI^68^.

We did not use much data thinning or methods to re-sample for autocorrelation yet, as Maxent often prefers^69^. But arguably, our research opens new science for these questions that have never been attempted yet on that scale. New insights can be expected counter to^70^. Arguably, we want to use a more fine-tuned, optimized, and complete workflow as well as more GIS habitat layers and an ensemble model in the future. Within bounds, additional Species Distribution Forecasts for future decades can be created using this workflow, not just for the global squirrel species but also for all other kinds of vertebrate species.

In summary, we found that the global squirrel hotspots are primarily located in North America, Europe, Central America, Northwestern Africa, Western Asia, most regions in Eastern Asia, Southeast Asia, and Tropical Africa. On the other hand, we found that the global squirrel coldspots are located in the North American Arctic, Greenland, parts of South America, Southwestern Africa, Sahara & Sahel desert (Africa), the Middle East, Siberia and Tibet, New Guinea, Australia & Oceania, and Antarctica.

Now that such Super SDM methods are developed with transparent and shared workflows and metadata, we encourage all SDM users to apply such methods rather than parsimonious approaches. In order for everyone to run such Super SDM methods, we conclude that more access to cloud computing should be provided to the wider public and the need for policy to use this work.

### Supplementary Information

Supplementary Information 1.

Supplementary Information 2.

Supplementary Information 3.

Supplementary Information 4.

Supplementary Information 5.

Supplementary Information 6.

Supplementary Legends.

## Data Availability

All data utilized for this study will become publicly available upon the publication of this study. All directly represented data can be accessed in the Appendix, and any other one can be obtained on request from M. Steiner.
