# Supplementary material for: With super SDMs (machine learning, open access big data, and the cloud) towards more holistic global squirrel hotspots and coldspots
Source: Sci Rep. 2024 Mar 3;14:5204. doi: 10.1038/s41598-024-55173-8 (PMC10909860; doi:10.1038/s41598-024-55173-8)
Supplement: Supplementary file 1 — Supplementary Information 1. [file 41598_2024_55173_MOESM1_ESM.docx]

RGBIF Data

This file provides an explanation how the species-occurrence data from GBIF.org has been obtained for all global extant squirrel species, how it has been optimized and compacted and how it will be used for future studies. This procedure requires the previously downloaded software R.

To obtain the requested data (this method is also applicable for any species/ family dataset in GBIF, in this case the global extant squirrel species, have been taken as example), the statistical software R has to opened.

The following code has been used to obtain the data from GBIF.org. It provides step-by-step explanation how it has been obtained in R.

###This following code provides download possibilities for any taxon family and species from the online data archive GBIF.org#

##For this demonstration, all global extant squirrel species have been taken as example#

#Firstly, the necessary package has to installed, which allows specific functions to obtain the requested data#

install.packages("rgbif")

#The downloaded package has to recalled for current use#

library("rgbif")

#Subsequently, it is crucial to obtain the assigned taxon key for the species/genus/family of interest#

Sciuridaekey <- name_backbone(name = "Sciuridae")

#obtained information: "usageKey: 9456"#

#To check how many occurrences are stored in the GBIF achieve, execute the following command#

occ_count(taxonKey = 9456)

#obtained information: "[1] 961362"#

#A possible example overview of the data can be obtained by#

SquirrelData <- occ_search(scientificName = "Sciuridae", limit = 20)

#To download the data execute the following command, note that all requests/ specifications of the dataset have to be in separate "pred(X)" form.#

#Due to privacy protection, the username and password have been generalized#

DownloadDataa <- occ_download(pred('taxonKey',9456), format = "SIMPLE_CSV", user ="gbif_username", pwd = "gbif_password", email = "moriz.steiner.work@gmail.com", curlopts = list())

#To check the download status of the previously executed command (useful for big datasets), execute:#

occ_download_meta (DownloadDataa)

#obtained information: "Status: SUCCESSFUL

Format: SIMPLE_CSV

Download key: 0035494-200613084148143

..."#

#Whenever the previous command indicated the download as "SUCCESSFUL", the data can be exported from R to any desired folder on your pc, by inserting the previously obtained Download Key in the following command#

occ_download_get("0035494-200613084148143", path ="C:\\Users\\Moriz Steiner\\Trash\\Downloads", overwrite = TRUE)

#The data has been successfully downloaded and exported to the desired location on your pc#

#Enjoy the data#

#In case someone is only interested in single species, instead of an entire family, the procedure is identical, with the only difference that the taxon key is different#

#An example has been shown below#

library("rgbif")

THkey <- name_backbone(name = "Tamiasciurus hudsonicus")

DownloadData <- occ_download(pred('taxonKey',2437282), format = "SIMPLE_CSV", user ="gbif_username", pwd = "gbif_password", email = "moriz.steiner.work@gmail.com", curlopts = list())

occ_download_get("0035492-200613084148143", path ="C:\\Users\\Moriz Steiner\\Trash\\Downloads", overwrite = TRUE)

occ_download_meta (DownloadData)

These previous steps in R allowed it to obtain the dataset and to export it into excel, where the data set will be cleaned now and optimized for modern use in geo-mapping software such as ArcGIS/ QGIS.

To clean the data set, it has to be opened in excel. There, the data can be found in a text format, which is unbeneficial for a clear overview. Therefore, the first step is to convert the text into columns, using the function “Text to Columns”. Subsequently, to eliminate unnecessary data and to lower the file size, unnecessary data columns can be removed (in this case we removed the columns where no, or very little data was available, such as “depth”, “depth accuracy”, “issue type”).

Subsequently, occurrences can be deleted where species name information is lacking. This can be done by selecting the row where the species names are listed, use the feature “Find and Select”, then “Go to special…”, select “Blanks” and “OK”. This can be repeated for occurrences missing the date or any other metrics used for future investigations. Here, we also removed all occurrences lacking geographical information (decimal latitude and longitude, since this data will later be mapped in ArcGIS or QGIS) since they cannot be mapped later on.

Further clean up and rearrangements can be done depending on the need for the data.

Enjoy your GBIF exported data.

Moriz Steiner
