# Supplementary material for: With super SDMs (machine learning, open access big data, and the cloud) towards more holistic global squirrel hotspots and coldspots
Source: Sci Rep. 2024 Mar 3;14:5204. doi: 10.1038/s41598-024-55173-8 (PMC10909860; doi:10.1038/s41598-024-55173-8)
Supplement: Supplementary file 2 — Supplementary Information 2. [file 41598_2024_55173_MOESM2_ESM.zip › MetadataBigDataOracleSquirrelColdspots_Vers1MS-summary.html]

Metadata Validation Service


### Validation and re-expression of 9e997b70.xml

```
mp 2.9.52 - Peter N. Schweitzer (U.S. Geological Survey)
89 errors: 2 misplaced, 87 empty, 3 warning
```

| Output | Description |
| --- | --- |
| Error report | Listing of errors and warnings, prioritized, color-coded, and linked to the relevant parts of the FGDC standard document. This page is generated by err2html. |
| Error listing | Raw text listing errors and warnings, directly from mp. |
| Questions and Answers | Metadata re-expressed as a set of frequently-anticipated questions, with answers drawn from the metadata record itself. See Metadata in Plain Language. |
| Outline | The metadata expressed in HTML using simple descriptive lists to show the record's structure. |
| Text | Indented text form of the metadata. |
| XML | Extensible Markup Language. Note that older web browsers will show this as a blank page, but you can see the actual document by choosing "Page Source" from the browser's "View" menu. |
| ZIP | A Zip package containing all of these files, renamed to match your original file name. |

These results will remain available for the rest of the day.

- DOI Privacy Policy
- Legal
- Accessibility
- Site Map
- Contact USGS

---

- U.S. Department of the Interior
- DOI Inspector General
- White House
- E-gov
- No Fear Act
- FOIA

- Follow
- *Twitter*
- *Facebook*
- *Google+*
- *GitHub*
- *Flickr*
- *YouTube*
- *Instagram*
