# Supplementary material for: With super SDMs (machine learning, open access big data, and the cloud) towards more holistic global squirrel hotspots and coldspots
Source: Sci Rep. 2024 Mar 3;14:5204. doi: 10.1038/s41598-024-55173-8 (PMC10909860; doi:10.1038/s41598-024-55173-8)
Supplement: Supplementary file 2 — Supplementary Information 2. [file 41598_2024_55173_MOESM2_ESM.zip › MetadataBigDataOracleSquirrelColdspots_Vers1MS.err.html]

Error report from mp


# mp 2.9.52 - Peter N. Schweitzer (U.S. Geological Survey)

89 errors: 2 misplaced, 87 empty, 3 warning

| Type | Description or line numbers | Line(s) (or count) |
| --- | --- | --- |
| Severity 2: Empty elements | | |
| Warning | Attribute\_Definition (5.1.2.2) does not have a value | (17) |
|  | 220, 254, 295, 322, 340, 348, 359, 376, 442, 508, 550, 615, 697, 779, 821, 898, 906 |  |
| Warning | Enumerated\_Domain\_Value\_Definition (5.1.2.4.1.2) does not have a value | (68) |
|  | 223, 231, 239, 247, 257, 265, 273, 281, 298, 306, 314, 379, 387, 395, 403, 411, 419, 427, 435, 445, 453, 461, 469, 477, 485, 493, 501, 511, 519, 527, 535, 543, 553, 561, 569, 577, 585, 593, 601, 618, 626, 634, 642, 650, 658, 666, 674, 682, 690, 700, 708, 716, 724, 732, 740, 748, 756, 764, 772, 782, 790, 798, 806, 814, 824, 832, 840, 848 |  |
| Warning | Unrepresentable\_Domain (5.1.2.4.4) does not have a value | 343 |
| Severity 0: Informative warnings and upgrade notes | | |
| Warning | Access\_Constraints (1.7) appears in unexpected order within Identification\_Information (1) | 118 |
| Warning | Bounding\_Coordinates (1.5.1) appears in unexpected order within Spatial\_Domain (1.5) | 34 |
| Error | Description\_of\_Geographic\_Extent (1.5.3) is not expected in Spatial\_Domain (1.5) | 33 |
| Other | Info: input file = phpBb6KNY.xml | 0 |
| Other | Info: process date = 20230331 | 0 |
| Other | Info: process time = 15:25:40 | 0 |
| Warning | Missing Spatial\_Reference\_Information (4) is mandatory if applicable in Metadata (0) | 2 |
| Error | Taxonomy is not expected in Identification\_Information (1) | 59 |

Generated by err2html 2.1.13
Fri Mar 31 15:25:40 2023
