# Supplementary material for: With super SDMs (machine learning, open access big data, and the cloud) towards more holistic global squirrel hotspots and coldspots
Source: Sci Rep. 2024 Mar 3;14:5204. doi: 10.1038/s41598-024-55173-8 (PMC10909860; doi:10.1038/s41598-024-55173-8)
Supplement: Supplementary file 2 — Supplementary Information 2. [file 41598_2024_55173_MOESM2_ESM.zip › MetadataBigDataOracleSquirrelColdspots_Vers1MS.faq.html]

Data for: With Super SDMs (Machine Learning, Open Access Big Data, and The Cloud) towards a more holistic and inclusive inference: Insights from progressing the marginalized case of the world’s squirrel hotspots and coldspots


# Data for: With Super SDMs (Machine Learning, Open Access Big Data, and The Cloud) towards a more holistic and inclusive inference: Insights from progressing the marginalized case of the world’s squirrel hotspots and coldspots

Metadata also available as - [Outline] - [Parseable text] - [XML]

#### Frequently anticipated questions:

- What does this data set describe?
  1. How might this data set be cited?
  2. What geographic area does the data set cover?
  3. What does it look like?
  4. Does the data set describe conditions during a particular time period?
  5. What is the general form of this data set?
  6. How does the data set represent geographic features?
  7. How does the data set describe geographic features?
  8. What biological taxa does this data set concern?
- Who produced the data set?
  1. Who are the originators of the data set?
  2. Who also contributed to the data set?
  3. To whom should users address questions about the data?
- Why was the data set created?
- How was the data set created?
  1. From what previous works were the data drawn?
  2. How were the data generated, processed, and modified?
  3. What similar or related data should the user be aware of?
- How reliable are the data; what problems remain in the data set?
  1. How well have the observations been checked?
  2. How accurate are the geographic locations?
  3. How accurate are the heights or depths?
  4. Where are the gaps in the data? What is missing?
  5. How consistent are the relationships among the data, including topology?
- How can someone get a copy of the data set?
  1. Are there legal restrictions on access or use of the data?
  2. Who distributes the data?
  3. What's the catalog number I need to order this data set?
  4. What legal disclaimers am I supposed to read?
  5. How can I download or order the data?
- Who wrote the metadata?

---

### What does this data set describe?

Title:

Data for: With Super SDMs (Machine Learning, Open Access Big Data, and The Cloud) towards a more holistic and inclusive inference: Insights from progressing the marginalized case of the world’s squirrel hotspots and coldspots

Abstract:

Species-habitat associations are correlative, can be quantified, and used for powerful inference. Nowadays, Species Distribution Models (SDMs) play a big role, e.g. using Machine Learning and AI algorithms, but their best-available technical opportunities remain still not used for their potential e.g. in the policy sector. Here we present Super SDMs that invoke ML, OA Big Data, and the Cloud with a workflow for the best-possible inference for the 300+ global squirrel species. Such global Big Data models are especially important for the many marginalized squirrel species and the high number of endangered and data-deficient species in the world, specifically in tropical regions. While our work shows common issues with SDMs and the maxent algorithm (‘Shallow Learning'), here we present a multi-species Big Data SDM template for subsequent ensemble models and generic progress to tackle global species hotspots and cold spots for the best possible outcome.

Supplemental\_Information:

The extensive Supplemental Information set for this study can also be accessed via the DOI provided here.

1. **How might this data set be cited?**

   Steiner, Moriz, and Huettmann, Falk, 20230331, Data for: With Super SDMs (Machine Learning, Open Access Big Data, and The Cloud) towards a more holistic and inclusive inference: Insights from progressing the marginalized case of the world’s squirrel hotspots and coldspots.
2. **What geographic area does the data set cover?**

   West\_Bounding\_Coordinate: -180.0000  
   East\_Bounding\_Coordinate: 180.0000  
   North\_Bounding\_Coordinate: 90.0000  
   South\_Bounding\_Coordinate: -90.0000  
   Description\_of\_Geographic\_Extent: World
3. **What does it look like?**
4. **Does the data set describe conditions during a particular time period?**

   Beginning\_Date: 01-Jan-2000

   Ending\_Date: 31-Dec-2021

   Currentness\_Reference:
   :   publication date
5. **What is the general form of this data set?**

   Geospatial\_Data\_Presentation\_Form: publication
6. **How does the data set represent geographic features?**
   1. **How are geographic features stored in the data set?**  
      This is a Raster data set.
      It contains the following raster data types:
      - Dimensions 4320 x 8640, type Pixel
   2. **What coordinate system is used to represent geographic features?**
7. **How does the data set describe geographic features?**

   Table1: Oracle cloud settings utilized for global squirrel SDM
   :   This table depicts the Oracle cloud settings used for the Super SDM.
       (Source: Producer Defined)

       Oracle cloud metric
       :   (Source: Producer Defined)

           | Value | Definition |
           | --- | --- |
           | Computer system |  |
           | CPU Capacity |  |
           | OCPU count |  |
           | Machine shape |  |

           Description
           :   (Source: Producer Defined)

               | Value | Definition |
               | --- | --- |
               | Linux |  |
               | 1024 GB |  |
               | 64 |  |
               | VM.Standard.E4.Flex |  |

       Table 2: Global squirrel Super SDM model evaluation
       :   This table depicts the model evaluation criteria of the global Super SDM.
           (Source: Producer Defined)

           Evaluation criteria
           :   (Source: Producer Defined)

               | Value | Definition |
               | --- | --- |
               | AUC (Area under the ROC Curve) |  |
               | Correlation |  |
               | Test accuracy |  |

               Description
               :   (Source: Producer Defined)

                   | Range of values | |
                   | --- | --- |
                   | Minimum: | 0.4198 |
                   | Maximum: | 0.9543 |

           Table 3: Global squirrel Super SDM variable importance
           :   This table depicts the variable importance of all included environmental predictors included in the Super SDM.
               (Source: Producer Defined)

               Variable
               :   (Source: Producer Defined)

                   Percent contribution
                   :   (Source: Producer Defined)

                       | Range of values | |
                       | --- | --- |
                       | Minimum: | 0.0 |
                       | Maximum: | 43.7 |

                       Permutation importance
                       :   (Source: Producer Defined)

                           | Range of values | |
                           | --- | --- |
                           | Minimum: | 0.0 |
                           | Maximum: | 30.8 |

               Table 4a: Global squirrel hotspot regions
               :   This table describes the global squirrel hotspot regions identified by the Super SDM.
                   (Source: Producer Defined)

                   Regions
                   :   (Source: Producer Defined)

                       | Value | Definition |
                       | --- | --- |
                       | North America |  |
                       | Europe |  |
                       | Central America |  |
                       | Northwestern Africa |  |
                       | Western Asia |  |
                       | Most eastern Asia |  |
                       | Southeast Asia |  |
                       | Tropical Africa |  |

                       Included countries
                       :   (Source: Producer Defined)

                           | Value | Definition |
                           | --- | --- |
                           | USA, Southern Canada |  |
                           | Portugal, Spain, United Kingdom, Ireland, France, Belgium, Netherlands, Germany, Denmark, Switzerland, Liechtenstein, Luxembourg, Austria, Italy, Slovenia, Poland, Sweden, Norway, Finland, Slovakia, Czechia, Hungary, Croatia, Romania, Serbia, Moldova, Ukraine, Bosnia and Herzegovina, Albania, Montenegro, Bulgaria, North Macedonia, Greece, Latvia, Lithuania, Estonia, Belarus |  |
                           | Mexico, Guatemala, Belize, Honduras, El Salvador, Nicaragua, Costa Rica, Cuba, Haiti, Dominican Republic, Puerto Rico, several island states |  |
                           | Morocco, North Algeria, Tunisia |  |
                           | Georgia, Armenia, Azerbaijan, Iran, Pakistan, Afghanistan, Turkmenistan, Tajikistan, Kyrgyzstan, Kazakhstan, Western Russia, Northern India, Nepal, Bhutan |  |
                           | South Korea, Japan, Taiwan |  |
                           | Vietnam, Thailand, Laos, Cambodia, Sri Lanka, Indonesia, Brunei, Malaysia, Philippines |  |
                           | Ethiopia, Western Kenya, Uganda, Rwanda, Burundi, Tanzania, Congo, DRC, Equatorial Guinea, Cameroon, South Sudan, Southwestern CAR, Nigeria, Benin, Togo, Burkina Faso, Ghana, Ivory Coast, Liberia, Sierra Leone, Guinea |  |

                           Reason(s) for high occurrences
                           :   (Source: Producer Defined)

                               | Value | Definition |
                               | --- | --- |
                               | Originating grounds (= long evolution time), close to Anthropocene (parks, bird feeders, etc.), temperate and optimal climate for mammals, plenty of habitat diversity, prey abundance. |  |
                               | Close to Anthropocene (parks, bird feeders, etc.), temperate and optimal climate for mammals, plenty of habitat diversity, prey abundance. |  |
                               | Pristine tropical habitats, extraordinary habitat diversity, high number of different possible ecological niches, prey abundance. |  |
                               | High human impact (benefits of living close to the Anthropocene), ideal for arid-loving species (predominately ground squirrels). |  |
                               | High habitat diversity with significant altitude changes. Hotpots observed often close to areas with high human impact. |  |

                               References
                               :   (Source: Producer Defined)

                                   | Value | Definition |
                                   | --- | --- |
                                   | Krauze‐Gryz et al. 2021; Luckett and Hartenberger 1985; Nelson et al. 2014; Pearson and Ruggiero 2001; Pineda-Munoz et al 2021; Stein 2002; Steiner and Huettmann in press |  |
                                   | Cervellini et al. 2021; Krauze‐Gryz et al. 2021; Pearson and Ruggiero 2001; Pineda-Munoz et al 2021; Steiner and Huettmann in press |  |
                                   | Bookbinder and Ledec 1995; Huettmann 2015; Morales-Marroquín et al. 2022; Reid 2006 |  |
                                   | Aulagnier 2016; Jacobson et al. 2019 |  |
                                   | Bizhanova et al. 2022; Jacobson et al. 2019; Regmi and Huettmann 2020 |  |
                                   | Procheş et al. 2021; Sodhi et al. 2004, 2006, 2009, 2010 |  |
                                   | Awodoyin et al. 2015; Couvreur et al. 2021; Mittermeier et al. 2011; Reid 1998 |  |

                   Table 4b: Global squirrel coldspot regions
                   :   This table describes the global squirrel hotspot regions identified by the Super SDM.
                       (Source: Producer Defined)

                       Regions
                       :   (Source: Producer Defined)

                           | Value | Definition |
                           | --- | --- |
                           | North American Arctic |  |
                           | Greenland |  |
                           | South America |  |
                           | Southwestern Africa |  |
                           | Sahara & Sahel desert (Africa) |  |
                           | Middle East |  |
                           | Siberia and Tibet |  |
                           | New Guinea |  |
                           | Australia & Oceania |  |
                           | Antarctica |  |

                           Included countries
                           :   (Source: Producer Defined)

                               | Value | Definition |
                               | --- | --- |
                               | Alaska (USA), Canada |  |
                               | Greenland |  |
                               | Southern Venezuela, Guyana, Suriname, French Guinea, Southwestern Colombia, Peru, Northeastern Brazil, Bolivia, Northern Chile, Argentina |  |
                               | Angola, Eswatini, Namibia |  |
                               | Central and Southern Algeria, Western Sahara, Mauritania, Northern Mali, Niger, Chad, Sudan, Libya, Southern Egypt |  |
                               | Southern and Northern Saudi Arabia, Western Oman, Eastern Yemen |  |
                               | Western China, Central and Eastern Russia |  |
                               | Indonesia, Western Papua New Guinea |  |
                               | Australia, New Zealand, Solomon Islands, New Caledonia, Fiji, Vanuatu, and several island states |  |
                               | Antarctica |  |

                               Reason(s) for low occurrences
                               :   (Source: Producer Defined)

                                   | Value | Definition |
                                   | --- | --- |
                                   | Unfavorable climate (too cold temperatures), low feed availability |  |
                                   | Few Squirrels have reached that far south throughout evolution |  |
                                   | Unfavorable climate (too hot temperatures, and too arid) |  |
                                   | Unfavorable climate (too hot temperatures, and too arid), low feed availability |  |
                                   | Squirrels did not reach these regions yet (see Wallace Line) |  |

                                   References
                                   :   (Source: Producer Defined)

                                       | Value | Definition |
                                       | --- | --- |
                                       | Mittermeier et al. 2011; Steiner and Huettmann in press |  |
                                       | Abreu-Jr et al. 2020; Hafner et al. 1994; Pečnerová and Martínková 2012; Steiner and Huettmann in press |  |
                                       | Hainsworth 1995; Mittermeier et al. 2011; Steiner and Huettmann in press |  |
                                       | Bacon et al. 2013; Steiner and Huettmann in press; Van Oosterzee 1997; White et al. 2021 |  |

                       Figure 1: Occurrence points of all global squirrel species (300+) utilized for the global squirrel SDM downloaded from www.GBIF.org
                       :   This figure depicts the occurrence points of all global squirrel species (300+) utilized for the global squirrel SDM downloaded from www.GBIF.org.
                           (Source: Producer Defined)

                           Figure 2: Methodological Workflow Global Super SDM
                           :   This figure depicts the methodological Workflow of the Global Super SDM.
                               (Source: Producer Defined)

                               Figure 3: Global Squirrel Species Distribution Model created with machine learning algorithms in the Oracle cloud computer
                               :   This figure depicts the global Squirrel Species Distribution Model created with machine learning algorithms in the Oracle cloud computer.
                                   (Source: Producer Defined)

                                   Appendix 1: R script to obtain GBIF occurrence points utilizing the RGBIF package.
                                   :   This appendix includes the R script to obtain GBIF occurrence points utilizing the RGBIF package.
                                       (Source: Producer Defined)

                                       Appendix 2: ISO-compliant metadata
                                       :   This appendix includes the
                                           (Source: Producer Defined)

                                           Appendix 3: Squirrel species list with occurrence record counts
                                           :   This appendix includes the squirrel species list with occurrence record counts.
                                               (Source: Producer Defined)

                                               Species names
                                               :   (Source: Producer Defined)

                                                   Count of species
                                                   :   (Source: Producer Defined)

                                                       | Range of values | |
                                                       | --- | --- |
                                                       | Minimum: | 1 |
                                                       | Maximum: | 665529 |

                                               Appendix 4: Environmental predictors description (Reproduced Table 3.2 from Steiner and Huettmann in press)
                                               :   This appendix includes the environmental predictors description (Reproduced Table 3.2 from Steiner and Huettmann in press).
                                                   (Source: Producer Defined)

                                                   Appendix 5: Documented R script from the Maxent Cloud computing run
                                                   :   This appendix includes the documented R script from the Maxent Cloud computing run.
                                                       (Source: Producer Defined)

                                                       Appendix 6: TIFF raster file of the produced global SDM
                                                       :   This appendix includes the TIFF raster file of the produced global SDM.
                                                           (Source: Producer Defined)
8. **What biological taxa does this data set concern?**

   Taxonomy:

   Keywords/Taxon:

   Taxonomic\_Keyword\_Thesaurus: None  
   Taxonomic\_Keywords: Sciuridae

   Taxonomic\_Classification:

   Taxon\_Rank\_Name: Kingdom  
   Taxon\_Rank\_Value: Animalia  
   Taxonomic\_Classification:

   Taxon\_Rank\_Name: Subkingdom  
   Taxon\_Rank\_Value: Bilateria  
   Taxonomic\_Classification:

   Taxon\_Rank\_Name: Infrakingdom  
   Taxon\_Rank\_Value: Deuterostomia  
   Taxonomic\_Classification:

   Taxon\_Rank\_Name: Phylum  
   Taxon\_Rank\_Value: Chordata  
   Taxonomic\_Classification:

   Taxon\_Rank\_Name: Subphylum  
   Taxon\_Rank\_Value: Vertebrata  
   Taxonomic\_Classification:

   Taxon\_Rank\_Name: Infraphylum  
   Taxon\_Rank\_Value: Gnathostomata  
   Taxonomic\_Classification:

   Taxon\_Rank\_Name: Superclass  
   Taxon\_Rank\_Value: Tetrapoda  
   Taxonomic\_Classification:

   Taxon\_Rank\_Name: Class  
   Taxon\_Rank\_Value: Mammalia  
   Taxonomic\_Classification:

   Taxon\_Rank\_Name: Subclass  
   Taxon\_Rank\_Value: Theria  
   Taxonomic\_Classification:

   Taxon\_Rank\_Name: Infraclass  
   Taxon\_Rank\_Value: Eutheria  
   Taxonomic\_Classification:

   Taxon\_Rank\_Name: Order  
   Taxon\_Rank\_Value: Rodentia  
   Taxonomic\_Classification:

   Taxon\_Rank\_Name: Suborder  
   Taxon\_Rank\_Value: Sciuromorpha  
   Taxonomic\_Classification:

   Taxon\_Rank\_Name: Family  
   Taxon\_Rank\_Value: Sciuridae  
   Applicable\_Common\_Name: TSN: 180135

---

### Who produced the data set?

1. **Who are the originators of the data set?** (may include formal authors, digital compilers, and editors)
   - Moriz Steiner- Falk Huettmann
2. **Who also contributed to the data set?**
3. **To whom should users address questions about the data?**

   Moriz Steiner

   -EWHALE Lab- Biology and Wildlife Department, Institute of Arctic Biology, Fairbanks University of Alaska Fairbanks (UAF), Fairbanks, AK, USA

   Dr. Daimerstrasse 2

   Sand in Taufers, Bozen/ Suedtirol   

   Italy

   +39 3493122232 (voice)

   moriz.steiner.work@gmail.com

---

### Why was the data set created?

This study's main aim was to create a Super Species distribution model for the global squirrel species with available GBIF data. This has been done by using 132 environmental predictors for all available squirrel species on GBIF.

---

### How was the data set created?

1. **From what previous works were the data drawn?**
2. **How were the data generated, processed, and modified?**

   Date: 31-Mar-2023 (process 1 of 5): We created a global SDM assessment of all the world’s squirrel species utilizing machine learning algorithms powered by cloud computing. This study builds upon a workflow and data previously introduced by Steiner and Huettmann (in press) and expands on that approach and workflow using almost three times as many new data. This workflow has been presented in Figure 2 below. To our knowledge, this presents the highest number of predictors and occurrence records ever used for one SDM (see Huettmann et al. 2018 for 80 predictors, and Sriram and Huettmann unpublished for 100, and for multi-species models see Steiner and Huettmann in press for over 130). This moves maxent from a simple ‘shallow-learning’ SDM algorithm into authentic data mining. We thus like to call it a Super SDM with the following method steps. Data sources used in this process: - Huettmann, F., Mi, C., & Guo, Y. (2018). ‘Batteries’ in machine learning: A first experimental assessment of inference for Siberian Crane Breeding Grounds in the Russian High Arctic Based on ‘Shaving’74 predictors. Machine Learning for Ecology and Sustainable Natural Resource Management, 163-184. Date: 31-Mar-2023 (process 2 of 5): Big Data: Occurrence dataWe utilized all publicly-available online GBIF occurrences for the family Sciuridae (= squirrels) with a cut-off date of November 13th 2022 (www.GBIF.org receives new data submissions ongoing and updates its sets monthly). An older version of this downloaded dataset was used by Steiner and Huettmann (in press) in 2020 but got significantly updated and now contains a total of 1,543,980 raw occurrence points (see download DOI: https://doi.org/10.15468/dl.2banfj). These occurrence points have been obtained from GBIF utilizing the RGBIF package in R (Chamberlain et al. 2021). The R script that has been utilized to obtain the occurrence points can be found in Appendix 1. After obtaining the occurrence data from RGBIF, we removed duplicates in the dataset in order to make it easier to handle the model run. There are different approaches to using ‘double locations’ as those are ‘true’ data (Humphries et al. 2018); however, maxent is not a true data mining algorithm and relies on parsimonious concepts creating its own pseudo-absences (Elith et al. 2020; Phillips et al. 2009). Arguably, for our objectives, the duplicated occurrence points have assumably little influence on the global SDMs when all occurrences are combined, which we decided to do in order to create the global hotspot/ coldspot analysis for all squirrel species. After removing duplicates (utilizing “removing duplicates” function in MS Excel), we also removed all records without a geographic location and a described species name (Hart-Davis 2010), after which the dataset has been saved as CSV file and imported in the data directory to be accessible for the cloud super-computer. This data preparation necessity sets it apart from more advanced and deep-learning methods such as boosting (TreeNet) or bagging workflow etc., that are better able to work with raw and messy data within which the corresponding Machine Learning algorithm seeks for patterns (Grillo et al. 2022; Mi et al. 2017). This resulted in 665,529 final occurrence points which have been mapped and presented in Figure 1 below; see Appendix 2 for ISO-compliant metadata describing this unique resource.Figure 1 shows the utilized occurrence points for this study, retrieved from GBIF.org. A detailed list of all included squirrel species and their corresponding record counts can be found in Appendix 3. Data sources used in this process: - Humphries, G., D.R. Magness and F. Huettmann (2018). Machine Learning for Ecology and Sustainable Natural Resource Management. Springer, Switzerland- Phillips, S. J., Dudík, M., Elith, J., Graham, C. H., Lehmann, A., Leathwick, J., & Ferrier, S. (2009). Sample selection bias and presence‐only distribution models: implications for background and pseudo‐absence data. Ecological applications, 19(1), 181-197.- Hart-Davis, G. (2010). Creating and Using Excel Database Tables. In Beginning Microsoft Office 2010 (pp. 393-411). Berkeley, CA: Apress.- Grillo, M., Huettmann, F., Guglielmo, L., & Schiaparelli, S. (2022). Three-Dimensional Quantification of Copepods Predictive Distributions in the Ross Sea: First Data Based on a Machine Learning Model Approach and Open Access (FAIR) Data. Diversity, 14(5), 355.- Mi, C., Huettmann, F., Guo, Y., Han, X., & Wen, L. (2017). Why choose Random Forest to predict rare species distribution with few samples in large undersampled areas? Three Asian crane species models provide supporting evidence. PeerJ, 5, e2849. Date: 31-Mar-2023 (process 3 of 5): Environmental predictorsHere, we utilized a total of 132 environmental predictors; a set that has been firstly partially compiled by Sriram and Huettmann (unpublished) and first presented as the world’s most complete socio-economic habitat predictor set by Steiner and Huettmann (in press). Here it has been re-utilized for this study. A detailed description of all predictors and their sources can be found in Appendix 4 (reproduced Table 3.2 from Steiner and Huettmann in press). Date: 31-Mar-2023 (process 4 of 5): Cloud modelingIn order to process the high quantities of data utilized for this study – point data and habitat layer data -, we performed all modeling steps in the Oracle cloud super-computer (www.oracle.com) using the R environment for easy reproducibility. Thanks to a computing grant to FH in 2022 we were able to use the ORACLE cloud; we used the settings depicted in Table 1.Utilizing the settings presented in Table 1, we then ran ‘remotely’ a newly created R script for the global Super SDM (see Appendix 5) in the Windows PowerShell software, virtually synchronized with the oracle cloud computer. This SDM has been created utilizing Maxent (version 3.4.4 – https://biodiversityinformatics.amnh.org/open\_source/maxent/) and the software packages “raster”, “dismo”, “rgeos”, “sp”, and “rJava” (see corresponding references in sequence of the included packages – Hijmans and van Etten 2016; Hijmans and Elith 2013; Bivand et al. 2017; Pebesma et al. 2012; Urbanek 2013). In order to subsequently produce the desired SDM, we ran the commands “maxent” and “predict” in Windows PowerShell. To diminish possible data gaps as much as possible, we utilized 80% of the available data for training the ML model and the remaining 20% and 500 iterations for the model testing. This ratio of data attributed to training and testing is commonly found in literature but many models use a ratio of data for the model training that is smaller (sometimes significantly smaller) than the model testing ratio (Joseph 2022). With our approach, we believe to have diminished possible data gaps as much as possible while still testing the model sufficiently with the remaining 20% of the data and 500 iterations. An overview of the workflow performed in this study is displayed in Figure 2. This workflow includes all steps performed in the creation of the Super SDM in this study. It starts with the collection of the required datasets and ends with the results of the SDM in GIS. Additional add-on options are also included in this workflow, e.g. the option to create ensemble models. This workflow can act as a template for future Super SDMs studies, assessing other vertebrate species. Data sources used in this process: - Hijmans, R. J., & van Etten, J. (2016). raster: Geographic data analysis and modeling. R package version, 2(8).- Hijmans, R. J., & Elith, J. (2013). Species distribution modeling with R. R Cran Project.- Bivand, R., Rundel, C., Pebesma, E., Stuetz, R., Hufthammer, K. O., & Bivand, M. R. (2017). Package ‘rgeos’. The Comprehensive R Archive Network (CRAN).- Pebesma, E., Bivand, R., Pebesma, M. E., RColorBrewer, S., & Collate, A. A. A. (2012). Package ‘sp’. The Comprehensive R Archive Network.- Urbanek, S. (2013). rJava: Low-level R to Java interface. http://www.rforge.net/rJava/- Joseph, V. R. (2022). Optimal ratio for data splitting. Statistical Analysis and Data Mining: The ASA Data Science Journal, 15(4), 531-538. Date: 31-Mar-2023 (process 5 of 5): Hotspot/ coldspot identificationOnce the SDM has been created, the produced raster has been imported into Open-Source GIS (QGIS version 3.10.6, obtainable via https://www.qgis.org/en/site/forusers/download.html); we also used ESRI ArcGIS for some operations. In GIS, with a visual rapid-assessment approach, we identified the global squirrel hotspots and coldspots. This distribution hotspot/ coldspot identification aims to show the predicted species distribution index of all global squirrel species (multi-species distribution index). Regions with a prediction index ≤ 0.32 have been classified as ‘coldspots’ (low prediction occurrence), and regions with a prediction index ≥ 0.66 have been classified as ‘hotspots’ (high prediction occurrence). These thresholds have been set up in this manner to represent the low 1/3rd of the predicted occurrence index as coldspots with the very little predicted occurrence, a certain average or medium, and the top 1/3rd of the predicted occurrence index as hotspots with very high predicted occurrences. Because our work is fully open access, any of these settings can be re-visited and improved upon new data and research.
3. **What similar or related data should the user be aware of?**

---

### How reliable are the data; what problems remain in the data set?

1. **How well have the observations been checked?**  
   The data that has been created for this study and dataset has the intention to be the most complete and state-of-the-art dataset on the species distribution of all global squirrel species. This was possible by using 132 environmental predictors obtained from Steiner and Huettmann (in press).
2. **How accurate are the geographic locations?**
3. **How accurate are the heights or depths?**
4. **Where are the gaps in the data? What is missing?**  
   This dataset is as complete as it was possible using open-access data in 2022 and 2023. Regarding the completeness of the raster dataset, we have included 132 environmental predictors of high quality on a global scale. This seems to be the most complete and holistic raster dataset in all published literature.
5. **How consistent are the relationships among the observations, including topology?**  
   The data matches well with all details provided.

---

### How can someone get a copy of the data set?

**Are there legal restrictions on access or use of the data?**

|  |  |
| --- | --- |
| Access\_Constraints | None. Please see 'Distribution Info' for details. |
| Use\_Constraints | None. Users are advised to read the dataset's metadata thoroughly to understand appropriate use and data limitations. |

1. **Who distributes the data set?** (Distributor 1 of 1)  

   Moriz Steiner

   EWHALE lab- Inst of Arctic Biology, Department of Conservation Ecology, UnEWHALE lab- Inst of Arctic Biology, Department of Conservation Ecology, University of Alaska Fairbanks, Fairbanks, AK, USA

   Dr. Daimerstrasse 2

   Sand in Taufers, Bozen/ Suedtirol   

   Italy

   +39 3493122232 (voice)

   moriz.steiner.work@gmail.com
2. **What's the catalog number I need to order this data set?**
3. **What legal disclaimers am I supposed to read?**

   Unless otherwise stated, all data, metadata and related materials are considered to satisfy the quality standards relative to the purpose for which the data were collected. Although these data and associated metadata have been reviewed for accuracy and completeness and approved for release by the U.S. Geological Survey (USGS), no warranty expressed or implied is made regarding the display or utility of the data on any other system or for general or scientific purposes, nor shall the act of distribution constitute any such warranty.
4. **How can I download or order the data?**

---

### Who wrote the metadata?

Dates:: Last modified: 31-Mar-2023 Metadata author:: Moriz Steiner -EWHALE Lab- Biology and Wildlife Department, Institute of Arctic Biology, Fairbanks University of Alaska Fairbanks (UAF), Fairbanks, AK, USA Dr. Daimerstrasse 2 Sand in Taufers, Bozen/ Suedtirol Italy +39 3493122232 (voice) moriz.steiner.work@gmail.com Metadata standard:: FGDC Biological Data Profile of the Content Standard for Digital Geospatial Metadata (FGDC-STD-001.1-1999)

---

Generated by mp version 2.9.52 on Fri Mar 31 15:25:40 2023
