# Supplementary material for: With super SDMs (machine learning, open access big data, and the cloud) towards more holistic global squirrel hotspots and coldspots
Source: Sci Rep. 2024 Mar 3;14:5204. doi: 10.1038/s41598-024-55173-8 (PMC10909860; doi:10.1038/s41598-024-55173-8)
Supplement: Supplementary file 2 — Supplementary Information 2. [file 41598_2024_55173_MOESM2_ESM.zip › MetadataBigDataOracleSquirrelColdspots_Vers1MS.html]

Data for: With Super SDMs (Machine Learning, Open Access Big Data, and The Cloud) towards a more holistic and inclusive inference: Insights from progressing the marginalized case of the world’s squirrel hotspots and coldspots


# Data for: With Super SDMs (Machine Learning, Open Access Big Data, and The Cloud) towards a more holistic and inclusive inference: Insights from progressing the marginalized case of the world’s squirrel hotspots and coldspots

Metadata also available as - [Questions & Answers] - [Parseable text] - [XML]

### Metadata:

- Identification\_Information
- Data\_Quality\_Information
- Spatial\_Data\_Organization\_Information
- Entity\_and\_Attribute\_Information
- Distribution\_Information
- Metadata\_Reference\_Information

Identification\_Information:

Citation:

Citation\_Information:

Originator: Moriz Steiner  
Originator: Falk Huettmann  
Publication\_Date: 20230331  
Title:

Data for: With Super SDMs (Machine Learning, Open Access Big Data, and The Cloud) towards a more holistic and inclusive inference: Insights from progressing the marginalized case of the world’s squirrel hotspots and coldspots

Edition: 1  
Geospatial\_Data\_Presentation\_Form: publication

Description:

Abstract:

Species-habitat associations are correlative, can be quantified, and used for powerful inference. Nowadays, Species Distribution Models (SDMs) play a big role, e.g. using Machine Learning and AI algorithms, but their best-available technical opportunities remain still not used for their potential e.g. in the policy sector. Here we present Super SDMs that invoke ML, OA Big Data, and the Cloud with a workflow for the best-possible inference for the 300+ global squirrel species. Such global Big Data models are especially important for the many marginalized squirrel species and the high number of endangered and data-deficient species in the world, specifically in tropical regions. While our work shows common issues with SDMs and the maxent algorithm (‘Shallow Learning'), here we present a multi-species Big Data SDM template for subsequent ensemble models and generic progress to tackle global species hotspots and cold spots for the best possible outcome.

Purpose:

This study's main aim was to create a Super Species distribution model for the global squirrel species with available GBIF data. This has been done by using 132 environmental predictors for all available squirrel species on GBIF.

Supplemental\_Information:

The extensive Supplemental Information set for this study can also be accessed via the DOI provided here.

Time\_Period\_of\_Content:

Time\_Period\_Information:

Range\_of\_Dates/Times:

Beginning\_Date: 20000101  
Ending\_Date: 20211231

Currentness\_Reference: publication date

Status:

Progress: Complete  
Maintenance\_and\_Update\_Frequency: As needed

Spatial\_Domain:

Description\_of\_Geographic\_Extent: World  
Bounding\_Coordinates:

West\_Bounding\_Coordinate: -180.0000  
East\_Bounding\_Coordinate: 180.0000  
North\_Bounding\_Coordinate: 90.0000  
South\_Bounding\_Coordinate: -90.0000

Keywords:

Theme:

Theme\_Keyword\_Thesaurus: ISO 19115 Topic Category  
Theme\_Keyword: biota

Theme:

Theme\_Keyword\_Thesaurus: None  
Theme\_Keyword: Squirrels  
Theme\_Keyword: Sciuridae  
Theme\_Keyword: BIG DATA  
Theme\_Keyword: MaxEnt  
Theme\_Keyword: Super Species Distribution Models  
Theme\_Keyword: R  
Theme\_Keyword: Oracle  
Theme\_Keyword: Super computer  
Theme\_Keyword: Cloud modeling

Taxonomy:

Keywords/Taxon:

Taxonomic\_Keyword\_Thesaurus: None  
Taxonomic\_Keywords: Sciuridae

Taxonomic\_Classification:

Taxon\_Rank\_Name: Kingdom  
Taxon\_Rank\_Value: Animalia  
Taxonomic\_Classification:

Taxon\_Rank\_Name: Subkingdom  
Taxon\_Rank\_Value: Bilateria  
Taxonomic\_Classification:

Taxon\_Rank\_Name: Infrakingdom  
Taxon\_Rank\_Value: Deuterostomia  
Taxonomic\_Classification:

Taxon\_Rank\_Name: Phylum  
Taxon\_Rank\_Value: Chordata  
Taxonomic\_Classification:

Taxon\_Rank\_Name: Subphylum  
Taxon\_Rank\_Value: Vertebrata  
Taxonomic\_Classification:

Taxon\_Rank\_Name: Infraphylum  
Taxon\_Rank\_Value: Gnathostomata  
Taxonomic\_Classification:

Taxon\_Rank\_Name: Superclass  
Taxon\_Rank\_Value: Tetrapoda  
Taxonomic\_Classification:

Taxon\_Rank\_Name: Class  
Taxon\_Rank\_Value: Mammalia  
Taxonomic\_Classification:

Taxon\_Rank\_Name: Subclass  
Taxon\_Rank\_Value: Theria  
Taxonomic\_Classification:

Taxon\_Rank\_Name: Infraclass  
Taxon\_Rank\_Value: Eutheria  
Taxonomic\_Classification:

Taxon\_Rank\_Name: Order  
Taxon\_Rank\_Value: Rodentia  
Taxonomic\_Classification:

Taxon\_Rank\_Name: Suborder  
Taxon\_Rank\_Value: Sciuromorpha  
Taxonomic\_Classification:

Taxon\_Rank\_Name: Family  
Taxon\_Rank\_Value: Sciuridae  
Applicable\_Common\_Name: TSN: 180135

Access\_Constraints: None. Please see 'Distribution Info' for details.  
Use\_Constraints:

None. Users are advised to read the dataset's metadata thoroughly to understand appropriate use and data limitations.

Point\_of\_Contact:

Contact\_Information:

Contact\_Person\_Primary:

Contact\_Person: Moriz Steiner  
Contact\_Organization:

-EWHALE Lab- Biology and Wildlife Department, Institute of Arctic Biology, Fairbanks University of Alaska Fairbanks (UAF), Fairbanks, AK, USA

Contact\_Address:

Address\_Type: mailing  
Address: Dr. Daimerstrasse 2  
City: Sand in Taufers  
State\_or\_Province: Bozen/ Suedtirol  
Postal\_Code: 39032  
Country: Italy

Contact\_Voice\_Telephone: +39 3493122232  
Contact\_Electronic\_Mail\_Address: moriz.steiner.work@gmail.com

Native\_Data\_Set\_Environment:

The main software tool used for the creation of this material are:
QGIS & ArcGIS = https://qgis.org/en/site/forusers/download.html & https://pro.arcgis.com/en/pro-app/latest/get-started/download-arcgis-pro.html
Maxent = https://biodiversityinformatics.amnh.org/open\_source/maxent/
R = https://cran.r-project.org/bin/windows/base/For all programs the latest versions have been used referenced to the publication date.

Data\_Quality\_Information:

Attribute\_Accuracy:

Attribute\_Accuracy\_Report:

The data that has been created for this study and dataset has the intention to be the most complete and state-of-the-art dataset on the species distribution of all global squirrel species. This was possible by using 132 environmental predictors obtained from Steiner and Huettmann (in press).

Logical\_Consistency\_Report: The data matches well with all details provided.  
Completeness\_Report:

This dataset is as complete as it was possible using open-access data in 2022 and 2023. Regarding the completeness of the raster dataset, we have included 132 environmental predictors of high quality on a global scale. This seems to be the most complete and holistic raster dataset in all published literature.

Lineage:

Process\_Step:

Process\_Description:

We created a global SDM assessment of all the world’s squirrel species utilizing machine learning algorithms powered by cloud computing. This study builds upon a workflow and data previously introduced by Steiner and Huettmann (in press) and expands on that approach and workflow using almost three times as many new data. This workflow has been presented in Figure 2 below. To our knowledge, this presents the highest number of predictors and occurrence records ever used for one SDM (see Huettmann et al. 2018 for 80 predictors, and Sriram and Huettmann unpublished for 100, and for multi-species models see Steiner and Huettmann in press for over 130). This moves maxent from a simple ‘shallow-learning’ SDM algorithm into authentic data mining. We thus like to call it a Super SDM with the following method steps.

Source\_Used\_Citation\_Abbreviation:

Huettmann, F., Mi, C., & Guo, Y. (2018). ‘Batteries’ in machine learning: A first experimental assessment of inference for Siberian Crane Breeding Grounds in the Russian High Arctic Based on ‘Shaving’74 predictors. Machine Learning for Ecology and Sustainable Natural Resource Management, 163-184.

Process\_Date: 20230331

Process\_Step:

Process\_Description:

Big Data: Occurrence dataWe utilized all publicly-available online GBIF occurrences for the family Sciuridae (= squirrels) with a cut-off date of November 13th 2022 (www.GBIF.org receives new data submissions ongoing and updates its sets monthly). An older version of this downloaded dataset was used by Steiner and Huettmann (in press) in 2020 but got significantly updated and now contains a total of 1,543,980 raw occurrence points (see download DOI: https://doi.org/10.15468/dl.2banfj). These occurrence points have been obtained from GBIF utilizing the RGBIF package in R (Chamberlain et al. 2021). The R script that has been utilized to obtain the occurrence points can be found in Appendix 1. After obtaining the occurrence data from RGBIF, we removed duplicates in the dataset in order to make it easier to handle the model run. There are different approaches to using ‘double locations’ as those are ‘true’ data (Humphries et al. 2018); however, maxent is not a true data mining algorithm and relies on parsimonious concepts creating its own pseudo-absences (Elith et al. 2020; Phillips et al. 2009). Arguably, for our objectives, the duplicated occurrence points have assumably little influence on the global SDMs when all occurrences are combined, which we decided to do in order to create the global hotspot/ coldspot analysis for all squirrel species. After removing duplicates (utilizing “removing duplicates” function in MS Excel), we also removed all records without a geographic location and a described species name (Hart-Davis 2010), after which the dataset has been saved as CSV file and imported in the data directory to be accessible for the cloud super-computer. This data preparation necessity sets it apart from more advanced and deep-learning methods such as boosting (TreeNet) or bagging workflow etc., that are better able to work with raw and messy data within which the corresponding Machine Learning algorithm seeks for patterns (Grillo et al. 2022; Mi et al. 2017). This resulted in 665,529 final occurrence points which have been mapped and presented in Figure 1 below; see Appendix 2 for ISO-compliant metadata describing this unique resource.Figure 1 shows the utilized occurrence points for this study, retrieved from GBIF.org. A detailed list of all included squirrel species and their corresponding record counts can be found in Appendix 3.

Source\_Used\_Citation\_Abbreviation:

Humphries, G., D.R. Magness and F. Huettmann (2018). Machine Learning for Ecology and Sustainable Natural Resource Management. Springer, Switzerland

Source\_Used\_Citation\_Abbreviation:

Phillips, S. J., Dudík, M., Elith, J., Graham, C. H., Lehmann, A., Leathwick, J., & Ferrier, S. (2009). Sample selection bias and presence‐only distribution models: implications for background and pseudo‐absence data. Ecological applications, 19(1), 181-197.

Source\_Used\_Citation\_Abbreviation:

Hart-Davis, G. (2010). Creating and Using Excel Database Tables. In Beginning Microsoft Office 2010 (pp. 393-411). Berkeley, CA: Apress.

Source\_Used\_Citation\_Abbreviation:

Grillo, M., Huettmann, F., Guglielmo, L., & Schiaparelli, S. (2022). Three-Dimensional Quantification of Copepods Predictive Distributions in the Ross Sea: First Data Based on a Machine Learning Model Approach and Open Access (FAIR) Data. Diversity, 14(5), 355.

Source\_Used\_Citation\_Abbreviation:

Mi, C., Huettmann, F., Guo, Y., Han, X., & Wen, L. (2017). Why choose Random Forest to predict rare species distribution with few samples in large undersampled areas? Three Asian crane species models provide supporting evidence. PeerJ, 5, e2849.

Process\_Date: 20230331

Process\_Step:

Process\_Description:

Environmental predictorsHere, we utilized a total of 132 environmental predictors; a set that has been firstly partially compiled by Sriram and Huettmann (unpublished) and first presented as the world’s most complete socio-economic habitat predictor set by Steiner and Huettmann (in press). Here it has been re-utilized for this study. A detailed description of all predictors and their sources can be found in Appendix 4 (reproduced Table 3.2 from Steiner and Huettmann in press).

Process\_Date: 20230331

Process\_Step:

Process\_Description:

Cloud modelingIn order to process the high quantities of data utilized for this study – point data and habitat layer data -, we performed all modeling steps in the Oracle cloud super-computer (www.oracle.com) using the R environment for easy reproducibility.
Thanks to a computing grant to FH in 2022 we were able to use the ORACLE cloud; we used the settings depicted in Table 1.Utilizing the settings presented in Table 1, we then ran ‘remotely’ a newly created R script for the global Super SDM (see Appendix 5) in the Windows PowerShell software, virtually synchronized with the oracle cloud computer. This SDM has been created utilizing Maxent (version 3.4.4 – https://biodiversityinformatics.amnh.org/open\_source/maxent/) and the software packages “raster”, “dismo”, “rgeos”, “sp”, and “rJava” (see corresponding references in sequence of the included packages – Hijmans and van Etten 2016; Hijmans and Elith 2013; Bivand et al. 2017; Pebesma et al. 2012; Urbanek 2013). In order to subsequently produce the desired SDM, we ran the commands “maxent” and “predict” in Windows PowerShell. To diminish possible data gaps as much as possible, we utilized 80% of the available data for training the ML model and the remaining 20% and 500 iterations for the model testing. This ratio of data attributed to training and testing is commonly found in literature but many models use a ratio of data for the model training that is smaller (sometimes significantly smaller) than the model testing ratio (Joseph 2022). With our approach, we believe to have diminished possible data gaps as much as possible while still testing the model sufficiently with the remaining 20% of the data and 500 iterations.
An overview of the workflow performed in this study is displayed in Figure 2. This workflow includes all steps performed in the creation of the Super SDM in this study. It starts with the collection of the required datasets and ends with the results of the SDM in GIS. Additional add-on options are also included in this workflow, e.g. the option to create ensemble models. This workflow can act as a template for future Super SDMs studies, assessing other vertebrate species.

Source\_Used\_Citation\_Abbreviation:

Hijmans, R. J., & van Etten, J. (2016). raster: Geographic data analysis and modeling. R package version, 2(8).

Source\_Used\_Citation\_Abbreviation:

Hijmans, R. J., & Elith, J. (2013). Species distribution modeling with R. R Cran Project.

Source\_Used\_Citation\_Abbreviation:

Bivand, R., Rundel, C., Pebesma, E., Stuetz, R., Hufthammer, K. O., & Bivand, M. R. (2017). Package ‘rgeos’. The Comprehensive R Archive Network (CRAN).

Source\_Used\_Citation\_Abbreviation:

Pebesma, E., Bivand, R., Pebesma, M. E., RColorBrewer, S., & Collate, A. A. A. (2012). Package ‘sp’. The Comprehensive R Archive Network.

Source\_Used\_Citation\_Abbreviation:

Urbanek, S. (2013). rJava: Low-level R to Java interface. http://www.rforge.net/rJava/

Source\_Used\_Citation\_Abbreviation:

Joseph, V. R. (2022). Optimal ratio for data splitting. Statistical Analysis and Data Mining: The ASA Data Science Journal, 15(4), 531-538.

Process\_Date: 20230331

Process\_Step:

Process\_Description:

Hotspot/ coldspot identificationOnce the SDM has been created, the produced raster has been imported into Open-Source GIS (QGIS version 3.10.6, obtainable via https://www.qgis.org/en/site/forusers/download.html); we also used ESRI ArcGIS for some operations. In GIS, with a visual rapid-assessment approach, we identified the global squirrel hotspots and coldspots. This distribution hotspot/ coldspot identification aims to show the predicted species distribution index of all global squirrel species (multi-species distribution index). Regions with a prediction index ≤ 0.32 have been classified as ‘coldspots’ (low prediction occurrence), and regions with a prediction index ≥ 0.66 have been classified as ‘hotspots’ (high prediction occurrence). These thresholds have been set up in this manner to represent the low 1/3rd of the predicted occurrence index as coldspots with the very little predicted occurrence, a certain average or medium, and the top 1/3rd of the predicted occurrence index as hotspots with very high predicted occurrences. Because our work is fully open access, any of these settings can be re-visited and improved upon new data and research.

Process\_Date: 20230331

Spatial\_Data\_Organization\_Information:

Direct\_Spatial\_Reference\_Method: Raster  
Raster\_Object\_Information:

Raster\_Object\_Type: Pixel  
Row\_Count: 4320  
Column\_Count: 8640

Entity\_and\_Attribute\_Information:

Detailed\_Description:

Entity\_Type:

Entity\_Type\_Label: Table1: Oracle cloud settings utilized for global squirrel SDM  
Entity\_Type\_Definition:

This table depicts the Oracle cloud settings used for the Super SDM.

Entity\_Type\_Definition\_Source: Producer Defined

Attribute:

Attribute\_Label: Oracle cloud metric  
Attribute\_Definition:Attribute\_Definition\_Source: Producer Defined  
Attribute\_Domain\_Values:

Enumerated\_Domain:

Enumerated\_Domain\_Value: Computer system  
Enumerated\_Domain\_Value\_Definition:Enumerated\_Domain\_Value\_Definition\_Source: Producer defined

Attribute\_Domain\_Values:

Enumerated\_Domain:

Enumerated\_Domain\_Value: CPU Capacity  
Enumerated\_Domain\_Value\_Definition:Enumerated\_Domain\_Value\_Definition\_Source: Producer defined

Attribute\_Domain\_Values:

Enumerated\_Domain:

Enumerated\_Domain\_Value: OCPU count  
Enumerated\_Domain\_Value\_Definition:Enumerated\_Domain\_Value\_Definition\_Source: Producer defined

Attribute\_Domain\_Values:

Enumerated\_Domain:

Enumerated\_Domain\_Value: Machine shape  
Enumerated\_Domain\_Value\_Definition:Enumerated\_Domain\_Value\_Definition\_Source: Producer defined

Attribute:

Attribute\_Label: Description  
Attribute\_Definition:Attribute\_Definition\_Source: Producer Defined  
Attribute\_Domain\_Values:

Enumerated\_Domain:

Enumerated\_Domain\_Value: Linux  
Enumerated\_Domain\_Value\_Definition:Enumerated\_Domain\_Value\_Definition\_Source: Producer defined

Attribute\_Domain\_Values:

Enumerated\_Domain:

Enumerated\_Domain\_Value: 1024 GB  
Enumerated\_Domain\_Value\_Definition:Enumerated\_Domain\_Value\_Definition\_Source: Producer defined

Attribute\_Domain\_Values:

Enumerated\_Domain:

Enumerated\_Domain\_Value: 64  
Enumerated\_Domain\_Value\_Definition:Enumerated\_Domain\_Value\_Definition\_Source: Producer defined

Attribute\_Domain\_Values:

Enumerated\_Domain:

Enumerated\_Domain\_Value: VM.Standard.E4.Flex  
Enumerated\_Domain\_Value\_Definition:Enumerated\_Domain\_Value\_Definition\_Source: Producer defined

Detailed\_Description:

Entity\_Type:

Entity\_Type\_Label: Table 2: Global squirrel Super SDM model evaluation  
Entity\_Type\_Definition:

This table depicts the model evaluation criteria of the global Super SDM.

Entity\_Type\_Definition\_Source: Producer Defined

Attribute:

Attribute\_Label: Evaluation criteria  
Attribute\_Definition:Attribute\_Definition\_Source: Producer Defined  
Attribute\_Domain\_Values:

Enumerated\_Domain:

Enumerated\_Domain\_Value: AUC (Area under the ROC Curve)  
Enumerated\_Domain\_Value\_Definition:Enumerated\_Domain\_Value\_Definition\_Source: Producer defined

Attribute\_Domain\_Values:

Enumerated\_Domain:

Enumerated\_Domain\_Value: Correlation  
Enumerated\_Domain\_Value\_Definition:Enumerated\_Domain\_Value\_Definition\_Source: Producer defined

Attribute\_Domain\_Values:

Enumerated\_Domain:

Enumerated\_Domain\_Value: Test accuracy  
Enumerated\_Domain\_Value\_Definition:Enumerated\_Domain\_Value\_Definition\_Source: Producer defined

Attribute:

Attribute\_Label: Description  
Attribute\_Definition:Attribute\_Definition\_Source: Producer Defined  
Attribute\_Domain\_Values:

Range\_Domain:

Range\_Domain\_Minimum: 0.4198  
Range\_Domain\_Maximum: 0.9543

Detailed\_Description:

Entity\_Type:

Entity\_Type\_Label: Table 3: Global squirrel Super SDM variable importance  
Entity\_Type\_Definition:

This table depicts the variable importance of all included environmental predictors included in the Super SDM.

Entity\_Type\_Definition\_Source: Producer Defined

Attribute:

Attribute\_Label: Variable  
Attribute\_Definition:Attribute\_Definition\_Source: Producer Defined  
Attribute\_Domain\_Values:

Unrepresentable\_Domain:

Attribute:

Attribute\_Label: Percent contribution  
Attribute\_Definition:Attribute\_Definition\_Source: Producer Defined  
Attribute\_Domain\_Values:

Range\_Domain:

Range\_Domain\_Minimum: 0.0  
Range\_Domain\_Maximum: 43.7

Attribute:

Attribute\_Label: Permutation importance  
Attribute\_Definition:Attribute\_Definition\_Source: Producer Defined  
Attribute\_Domain\_Values:

Range\_Domain:

Range\_Domain\_Minimum: 0.0  
Range\_Domain\_Maximum: 30.8

Detailed\_Description:

Entity\_Type:

Entity\_Type\_Label: Table 4a: Global squirrel hotspot regions  
Entity\_Type\_Definition:

This table describes the global squirrel hotspot regions identified by the Super SDM.

Entity\_Type\_Definition\_Source: Producer Defined

Attribute:

Attribute\_Label: Regions  
Attribute\_Definition:Attribute\_Definition\_Source: Producer Defined  
Attribute\_Domain\_Values:

Enumerated\_Domain:

Enumerated\_Domain\_Value: North America  
Enumerated\_Domain\_Value\_Definition:Enumerated\_Domain\_Value\_Definition\_Source: Producer defined

Attribute\_Domain\_Values:

Enumerated\_Domain:

Enumerated\_Domain\_Value: Europe  
Enumerated\_Domain\_Value\_Definition:Enumerated\_Domain\_Value\_Definition\_Source: Producer defined

Attribute\_Domain\_Values:

Enumerated\_Domain:

Enumerated\_Domain\_Value: Central America  
Enumerated\_Domain\_Value\_Definition:Enumerated\_Domain\_Value\_Definition\_Source: Producer defined

Attribute\_Domain\_Values:

Enumerated\_Domain:

Enumerated\_Domain\_Value: Northwestern Africa  
Enumerated\_Domain\_Value\_Definition:Enumerated\_Domain\_Value\_Definition\_Source: Producer defined

Attribute\_Domain\_Values:

Enumerated\_Domain:

Enumerated\_Domain\_Value: Western Asia  
Enumerated\_Domain\_Value\_Definition:Enumerated\_Domain\_Value\_Definition\_Source: Producer defined

Attribute\_Domain\_Values:

Enumerated\_Domain:

Enumerated\_Domain\_Value: Most eastern Asia  
Enumerated\_Domain\_Value\_Definition:Enumerated\_Domain\_Value\_Definition\_Source: Producer defined

Attribute\_Domain\_Values:

Enumerated\_Domain:

Enumerated\_Domain\_Value: Southeast Asia  
Enumerated\_Domain\_Value\_Definition:Enumerated\_Domain\_Value\_Definition\_Source: Producer defined

Attribute\_Domain\_Values:

Enumerated\_Domain:

Enumerated\_Domain\_Value: Tropical Africa  
Enumerated\_Domain\_Value\_Definition:Enumerated\_Domain\_Value\_Definition\_Source: Producer defined

Attribute:

Attribute\_Label: Included countries  
Attribute\_Definition:Attribute\_Definition\_Source: Producer Defined  
Attribute\_Domain\_Values:

Enumerated\_Domain:

Enumerated\_Domain\_Value: USA, Southern Canada  
Enumerated\_Domain\_Value\_Definition:Enumerated\_Domain\_Value\_Definition\_Source: Producer defined

Attribute\_Domain\_Values:

Enumerated\_Domain:

Enumerated\_Domain\_Value:

Portugal, Spain, United Kingdom, Ireland, France, Belgium, Netherlands, Germany, Denmark, Switzerland, Liechtenstein, Luxembourg, Austria, Italy, Slovenia, Poland, Sweden, Norway, Finland, Slovakia, Czechia, Hungary, Croatia, Romania, Serbia, Moldova, Ukraine, Bosnia and Herzegovina, Albania, Montenegro, Bulgaria, North Macedonia, Greece, Latvia, Lithuania, Estonia, Belarus

Enumerated\_Domain\_Value\_Definition:Enumerated\_Domain\_Value\_Definition\_Source: Producer defined

Attribute\_Domain\_Values:

Enumerated\_Domain:

Enumerated\_Domain\_Value:

Mexico, Guatemala, Belize, Honduras, El Salvador, Nicaragua, Costa Rica, Cuba, Haiti, Dominican Republic, Puerto Rico, several island states

Enumerated\_Domain\_Value\_Definition:Enumerated\_Domain\_Value\_Definition\_Source: Producer defined

Attribute\_Domain\_Values:

Enumerated\_Domain:

Enumerated\_Domain\_Value: Morocco, North Algeria, Tunisia  
Enumerated\_Domain\_Value\_Definition:Enumerated\_Domain\_Value\_Definition\_Source: Producer defined

Attribute\_Domain\_Values:

Enumerated\_Domain:

Enumerated\_Domain\_Value:

Georgia, Armenia, Azerbaijan, Iran, Pakistan, Afghanistan, Turkmenistan, Tajikistan, Kyrgyzstan, Kazakhstan, Western Russia, Northern India, Nepal, Bhutan

Enumerated\_Domain\_Value\_Definition:Enumerated\_Domain\_Value\_Definition\_Source: Producer defined

Attribute\_Domain\_Values:

Enumerated\_Domain:

Enumerated\_Domain\_Value: South Korea, Japan, Taiwan  
Enumerated\_Domain\_Value\_Definition:Enumerated\_Domain\_Value\_Definition\_Source: Producer defined

Attribute\_Domain\_Values:

Enumerated\_Domain:

Enumerated\_Domain\_Value:

Vietnam, Thailand, Laos, Cambodia, Sri Lanka, Indonesia, Brunei, Malaysia, Philippines

Enumerated\_Domain\_Value\_Definition:Enumerated\_Domain\_Value\_Definition\_Source: Producer defined

Attribute\_Domain\_Values:

Enumerated\_Domain:

Enumerated\_Domain\_Value:

Ethiopia, Western Kenya, Uganda, Rwanda, Burundi, Tanzania, Congo, DRC, Equatorial Guinea, Cameroon, South Sudan, Southwestern CAR, Nigeria, Benin, Togo, Burkina Faso, Ghana, Ivory Coast, Liberia, Sierra Leone, Guinea

Enumerated\_Domain\_Value\_Definition:Enumerated\_Domain\_Value\_Definition\_Source: Producer defined

Attribute:

Attribute\_Label: Reason(s) for high occurrences  
Attribute\_Definition:Attribute\_Definition\_Source: Producer Defined  
Attribute\_Domain\_Values:

Enumerated\_Domain:

Enumerated\_Domain\_Value:

Originating grounds (= long evolution time), close to Anthropocene (parks, bird feeders, etc.), temperate and optimal climate for mammals, plenty of habitat diversity, prey abundance.

Enumerated\_Domain\_Value\_Definition:Enumerated\_Domain\_Value\_Definition\_Source: Producer defined

Attribute\_Domain\_Values:

Enumerated\_Domain:

Enumerated\_Domain\_Value:

Close to Anthropocene (parks, bird feeders, etc.), temperate and optimal climate for mammals, plenty of habitat diversity, prey abundance.

Enumerated\_Domain\_Value\_Definition:Enumerated\_Domain\_Value\_Definition\_Source: Producer defined

Attribute\_Domain\_Values:

Enumerated\_Domain:

Enumerated\_Domain\_Value:

Pristine tropical habitats, extraordinary habitat diversity, high number of different possible ecological niches, prey abundance.

Enumerated\_Domain\_Value\_Definition:Enumerated\_Domain\_Value\_Definition\_Source: Producer defined

Attribute\_Domain\_Values:

Enumerated\_Domain:

Enumerated\_Domain\_Value:

High human impact (benefits of living close to the Anthropocene), ideal for arid-loving species (predominately ground squirrels).

Enumerated\_Domain\_Value\_Definition:Enumerated\_Domain\_Value\_Definition\_Source: Producer defined

Attribute\_Domain\_Values:

Enumerated\_Domain:

Enumerated\_Domain\_Value:

High habitat diversity with significant altitude changes. Hotpots observed often close to areas with high human impact.

Enumerated\_Domain\_Value\_Definition:Enumerated\_Domain\_Value\_Definition\_Source: Producer defined

Attribute:

Attribute\_Label: References  
Attribute\_Definition:Attribute\_Definition\_Source: Producer Defined  
Attribute\_Domain\_Values:

Enumerated\_Domain:

Enumerated\_Domain\_Value:

Krauze‐Gryz et al. 2021; Luckett and Hartenberger 1985; Nelson et al. 2014; Pearson and Ruggiero 2001; Pineda-Munoz et al 2021; Stein 2002; Steiner and Huettmann in press

Enumerated\_Domain\_Value\_Definition:Enumerated\_Domain\_Value\_Definition\_Source: Producer defined

Attribute\_Domain\_Values:

Enumerated\_Domain:

Enumerated\_Domain\_Value:

Cervellini et al. 2021; Krauze‐Gryz et al. 2021; Pearson and Ruggiero 2001; Pineda-Munoz et al 2021; Steiner and Huettmann in press

Enumerated\_Domain\_Value\_Definition:Enumerated\_Domain\_Value\_Definition\_Source: Producer defined

Attribute\_Domain\_Values:

Enumerated\_Domain:

Enumerated\_Domain\_Value:

Bookbinder and Ledec 1995; Huettmann 2015; Morales-Marroquín et al. 2022; Reid 2006

Enumerated\_Domain\_Value\_Definition:Enumerated\_Domain\_Value\_Definition\_Source: Producer defined

Attribute\_Domain\_Values:

Enumerated\_Domain:

Enumerated\_Domain\_Value: Aulagnier 2016; Jacobson et al. 2019  
Enumerated\_Domain\_Value\_Definition:Enumerated\_Domain\_Value\_Definition\_Source: Producer defined

Attribute\_Domain\_Values:

Enumerated\_Domain:

Enumerated\_Domain\_Value:

Bizhanova et al. 2022; Jacobson et al. 2019; Regmi and Huettmann 2020

Enumerated\_Domain\_Value\_Definition:Enumerated\_Domain\_Value\_Definition\_Source: Producer defined

Attribute\_Domain\_Values:

Enumerated\_Domain:

Enumerated\_Domain\_Value: Procheş et al. 2021; Sodhi et al. 2004, 2006, 2009, 2010  
Enumerated\_Domain\_Value\_Definition:Enumerated\_Domain\_Value\_Definition\_Source: Producer defined

Attribute\_Domain\_Values:

Enumerated\_Domain:

Enumerated\_Domain\_Value:

Awodoyin et al. 2015; Couvreur et al. 2021; Mittermeier et al. 2011; Reid 1998

Enumerated\_Domain\_Value\_Definition:Enumerated\_Domain\_Value\_Definition\_Source: Producer defined

Detailed\_Description:

Entity\_Type:

Entity\_Type\_Label: Table 4b: Global squirrel coldspot regions  
Entity\_Type\_Definition:

This table describes the global squirrel hotspot regions identified by the Super SDM.

Entity\_Type\_Definition\_Source: Producer Defined

Attribute:

Attribute\_Label: Regions  
Attribute\_Definition:Attribute\_Definition\_Source: Producer Defined  
Attribute\_Domain\_Values:

Enumerated\_Domain:

Enumerated\_Domain\_Value: North American Arctic  
Enumerated\_Domain\_Value\_Definition:Enumerated\_Domain\_Value\_Definition\_Source: Producer defined

Attribute\_Domain\_Values:

Enumerated\_Domain:

Enumerated\_Domain\_Value: Greenland  
Enumerated\_Domain\_Value\_Definition:Enumerated\_Domain\_Value\_Definition\_Source: Producer defined

Attribute\_Domain\_Values:

Enumerated\_Domain:

Enumerated\_Domain\_Value: South America  
Enumerated\_Domain\_Value\_Definition:Enumerated\_Domain\_Value\_Definition\_Source: Producer defined

Attribute\_Domain\_Values:

Enumerated\_Domain:

Enumerated\_Domain\_Value: Southwestern Africa  
Enumerated\_Domain\_Value\_Definition:Enumerated\_Domain\_Value\_Definition\_Source: Producer defined

Attribute\_Domain\_Values:

Enumerated\_Domain:

Enumerated\_Domain\_Value: Sahara & Sahel desert (Africa)  
Enumerated\_Domain\_Value\_Definition:Enumerated\_Domain\_Value\_Definition\_Source: Producer defined

Attribute\_Domain\_Values:

Enumerated\_Domain:

Enumerated\_Domain\_Value: Middle East  
Enumerated\_Domain\_Value\_Definition:Enumerated\_Domain\_Value\_Definition\_Source: Producer defined

Attribute\_Domain\_Values:

Enumerated\_Domain:

Enumerated\_Domain\_Value: Siberia and Tibet  
Enumerated\_Domain\_Value\_Definition:Enumerated\_Domain\_Value\_Definition\_Source: Producer defined

Attribute\_Domain\_Values:

Enumerated\_Domain:

Enumerated\_Domain\_Value: New Guinea  
Enumerated\_Domain\_Value\_Definition:Enumerated\_Domain\_Value\_Definition\_Source: Producer defined

Attribute\_Domain\_Values:

Enumerated\_Domain:

Enumerated\_Domain\_Value: Australia & Oceania  
Enumerated\_Domain\_Value\_Definition:Enumerated\_Domain\_Value\_Definition\_Source: Producer defined

Attribute\_Domain\_Values:

Enumerated\_Domain:

Enumerated\_Domain\_Value: Antarctica  
Enumerated\_Domain\_Value\_Definition:Enumerated\_Domain\_Value\_Definition\_Source: Producer defined

Attribute:

Attribute\_Label: Included countries  
Attribute\_Definition:Attribute\_Definition\_Source: Producer Defined  
Attribute\_Domain\_Values:

Enumerated\_Domain:

Enumerated\_Domain\_Value: Alaska (USA), Canada  
Enumerated\_Domain\_Value\_Definition:Enumerated\_Domain\_Value\_Definition\_Source: Producer defined

Attribute\_Domain\_Values:

Enumerated\_Domain:

Enumerated\_Domain\_Value: Greenland  
Enumerated\_Domain\_Value\_Definition:Enumerated\_Domain\_Value\_Definition\_Source: Producer defined

Attribute\_Domain\_Values:

Enumerated\_Domain:

Enumerated\_Domain\_Value:

Southern Venezuela, Guyana, Suriname, French Guinea, Southwestern Colombia, Peru, Northeastern Brazil, Bolivia, Northern Chile, Argentina

Enumerated\_Domain\_Value\_Definition:Enumerated\_Domain\_Value\_Definition\_Source: Producer defined

Attribute\_Domain\_Values:

Enumerated\_Domain:

Enumerated\_Domain\_Value: Angola, Eswatini, Namibia  
Enumerated\_Domain\_Value\_Definition:Enumerated\_Domain\_Value\_Definition\_Source: Producer defined

Attribute\_Domain\_Values:

Enumerated\_Domain:

Enumerated\_Domain\_Value:

Central and Southern Algeria, Western Sahara, Mauritania, Northern Mali, Niger, Chad, Sudan, Libya, Southern Egypt

Enumerated\_Domain\_Value\_Definition:Enumerated\_Domain\_Value\_Definition\_Source: Producer defined

Attribute\_Domain\_Values:

Enumerated\_Domain:

Enumerated\_Domain\_Value: Southern and Northern Saudi Arabia, Western Oman, Eastern Yemen  
Enumerated\_Domain\_Value\_Definition:Enumerated\_Domain\_Value\_Definition\_Source: Producer defined

Attribute\_Domain\_Values:

Enumerated\_Domain:

Enumerated\_Domain\_Value: Western China, Central and Eastern Russia  
Enumerated\_Domain\_Value\_Definition:Enumerated\_Domain\_Value\_Definition\_Source: Producer defined

Attribute\_Domain\_Values:

Enumerated\_Domain:

Enumerated\_Domain\_Value: Indonesia, Western Papua New Guinea  
Enumerated\_Domain\_Value\_Definition:Enumerated\_Domain\_Value\_Definition\_Source: Producer defined

Attribute\_Domain\_Values:

Enumerated\_Domain:

Enumerated\_Domain\_Value:

Australia, New Zealand, Solomon Islands, New Caledonia, Fiji, Vanuatu, and several island states

Enumerated\_Domain\_Value\_Definition:Enumerated\_Domain\_Value\_Definition\_Source: Producer defined

Attribute\_Domain\_Values:

Enumerated\_Domain:

Enumerated\_Domain\_Value: Antarctica  
Enumerated\_Domain\_Value\_Definition:Enumerated\_Domain\_Value\_Definition\_Source: Producer defined

Attribute:

Attribute\_Label: Reason(s) for low occurrences  
Attribute\_Definition:Attribute\_Definition\_Source: Producer Defined  
Attribute\_Domain\_Values:

Enumerated\_Domain:

Enumerated\_Domain\_Value:

Unfavorable climate (too cold temperatures), low feed availability

Enumerated\_Domain\_Value\_Definition:Enumerated\_Domain\_Value\_Definition\_Source: Producer defined

Attribute\_Domain\_Values:

Enumerated\_Domain:

Enumerated\_Domain\_Value: Few Squirrels have reached that far south throughout evolution  
Enumerated\_Domain\_Value\_Definition:Enumerated\_Domain\_Value\_Definition\_Source: Producer defined

Attribute\_Domain\_Values:

Enumerated\_Domain:

Enumerated\_Domain\_Value: Unfavorable climate (too hot temperatures, and too arid)  
Enumerated\_Domain\_Value\_Definition:Enumerated\_Domain\_Value\_Definition\_Source: Producer defined

Attribute\_Domain\_Values:

Enumerated\_Domain:

Enumerated\_Domain\_Value:

Unfavorable climate (too hot temperatures, and too arid), low feed availability

Enumerated\_Domain\_Value\_Definition:Enumerated\_Domain\_Value\_Definition\_Source: Producer defined

Attribute\_Domain\_Values:

Enumerated\_Domain:

Enumerated\_Domain\_Value: Squirrels did not reach these regions yet (see Wallace Line)  
Enumerated\_Domain\_Value\_Definition:Enumerated\_Domain\_Value\_Definition\_Source: Producer defined

Attribute:

Attribute\_Label: References  
Attribute\_Definition:Attribute\_Definition\_Source: Producer Defined  
Attribute\_Domain\_Values:

Enumerated\_Domain:

Enumerated\_Domain\_Value: Mittermeier et al. 2011; Steiner and Huettmann in press  
Enumerated\_Domain\_Value\_Definition:Enumerated\_Domain\_Value\_Definition\_Source: Producer defined

Attribute\_Domain\_Values:

Enumerated\_Domain:

Enumerated\_Domain\_Value:

Abreu-Jr et al. 2020; Hafner et al. 1994; Pečnerová and Martínková 2012; Steiner and Huettmann in press

Enumerated\_Domain\_Value\_Definition:Enumerated\_Domain\_Value\_Definition\_Source: Producer defined

Attribute\_Domain\_Values:

Enumerated\_Domain:

Enumerated\_Domain\_Value:

Hainsworth 1995; Mittermeier et al. 2011; Steiner and Huettmann in press

Enumerated\_Domain\_Value\_Definition:Enumerated\_Domain\_Value\_Definition\_Source: Producer defined

Attribute\_Domain\_Values:

Enumerated\_Domain:

Enumerated\_Domain\_Value:

Bacon et al. 2013; Steiner and Huettmann in press; Van Oosterzee 1997; White et al. 2021

Enumerated\_Domain\_Value\_Definition:Enumerated\_Domain\_Value\_Definition\_Source: Producer defined

Detailed\_Description:

Entity\_Type:

Entity\_Type\_Label:

Figure 1: Occurrence points of all global squirrel species (300+) utilized for the global squirrel SDM downloaded from www.GBIF.org

Entity\_Type\_Definition:

This figure depicts the occurrence points of all global squirrel species (300+) utilized for the global squirrel SDM downloaded from www.GBIF.org.

Entity\_Type\_Definition\_Source: Producer Defined

Detailed\_Description:

Entity\_Type:

Entity\_Type\_Label: Figure 2: Methodological Workflow Global Super SDM  
Entity\_Type\_Definition:

This figure depicts the methodological Workflow of the Global Super SDM.

Entity\_Type\_Definition\_Source: Producer Defined

Detailed\_Description:

Entity\_Type:

Entity\_Type\_Label:

Figure 3: Global Squirrel Species Distribution Model created with machine learning algorithms in the Oracle cloud computer

Entity\_Type\_Definition:

This figure depicts the global Squirrel Species Distribution Model created with machine learning algorithms in the Oracle cloud computer.

Entity\_Type\_Definition\_Source: Producer Defined

Detailed\_Description:

Entity\_Type:

Entity\_Type\_Label:

Appendix 1: R script to obtain GBIF occurrence points utilizing the RGBIF package.

Entity\_Type\_Definition:

This appendix includes the R script to obtain GBIF occurrence points utilizing the RGBIF package.

Entity\_Type\_Definition\_Source: Producer Defined

Detailed\_Description:

Entity\_Type:

Entity\_Type\_Label: Appendix 2: ISO-compliant metadata  
Entity\_Type\_Definition: This appendix includes the  
Entity\_Type\_Definition\_Source: Producer Defined

Detailed\_Description:

Entity\_Type:

Entity\_Type\_Label: Appendix 3: Squirrel species list with occurrence record counts  
Entity\_Type\_Definition:

This appendix includes the squirrel species list with occurrence record counts.

Entity\_Type\_Definition\_Source: Producer Defined

Attribute:

Attribute\_Label: Species names  
Attribute\_Definition:Attribute\_Definition\_Source: Producer Defined  
Attribute\_Domain\_Values:

Unrepresentable\_Domain:

Attribute:

Attribute\_Label: Count of species  
Attribute\_Definition:Attribute\_Definition\_Source: Producer Defined  
Attribute\_Domain\_Values:

Range\_Domain:

Range\_Domain\_Minimum: 1  
Range\_Domain\_Maximum: 665529

Detailed\_Description:

Entity\_Type:

Entity\_Type\_Label:

Appendix 4: Environmental predictors description (Reproduced Table 3.2 from Steiner and Huettmann in press)

Entity\_Type\_Definition:

This appendix includes the environmental predictors description (Reproduced Table 3.2 from Steiner and Huettmann in press).

Entity\_Type\_Definition\_Source: Producer Defined

Detailed\_Description:

Entity\_Type:

Entity\_Type\_Label:

Appendix 5: Documented R script from the Maxent Cloud computing run

Entity\_Type\_Definition:

This appendix includes the documented R script from the Maxent Cloud computing run.

Entity\_Type\_Definition\_Source: Producer Defined

Detailed\_Description:

Entity\_Type:

Entity\_Type\_Label: Appendix 6: TIFF raster file of the produced global SDM  
Entity\_Type\_Definition:

This appendix includes the TIFF raster file of the produced global SDM.

Entity\_Type\_Definition\_Source: Producer Defined

Distribution\_Information:

Distributor:

Contact\_Information:

Contact\_Person\_Primary:

Contact\_Person: Moriz Steiner  
Contact\_Organization:

EWHALE lab- Inst of Arctic Biology, Department of Conservation Ecology, UnEWHALE lab- Inst of Arctic Biology, Department of Conservation Ecology, University of Alaska Fairbanks, Fairbanks, AK, USA

Contact\_Address:

Address\_Type: mailing address  
Address: Dr. Daimerstrasse 2  
City: Sand in Taufers  
State\_or\_Province: Bozen/ Suedtirol  
Postal\_Code: 39032  
Country: Italy

Contact\_Voice\_Telephone: +39 3493122232  
Contact\_Electronic\_Mail\_Address: moriz.steiner.work@gmail.com

Distribution\_Liability:

Unless otherwise stated, all data, metadata and related materials are considered to satisfy the quality standards relative to the purpose for which the data were collected. Although these data and associated metadata have been reviewed for accuracy and completeness and approved for release by the U.S. Geological Survey (USGS), no warranty expressed or implied is made regarding the display or utility of the data on any other system or for general or scientific purposes, nor shall the act of distribution constitute any such warranty.

Metadata\_Reference\_Information:

Metadata\_Date: 20230331  
Metadata\_Contact:

Contact\_Information:

Contact\_Person\_Primary:

Contact\_Person: Moriz Steiner  
Contact\_Organization:

-EWHALE Lab- Biology and Wildlife Department, Institute of Arctic Biology, Fairbanks University of Alaska Fairbanks (UAF), Fairbanks, AK, USA

Contact\_Address:

Address\_Type: mailing  
Address: Dr. Daimerstrasse 2  
City: Sand in Taufers  
State\_or\_Province: Bozen/ Suedtirol  
Postal\_Code: 39032  
Country: Italy

Contact\_Voice\_Telephone: +39 3493122232  
Contact\_Electronic\_Mail\_Address: moriz.steiner.work@gmail.com

Metadata\_Standard\_Name:

FGDC Biological Data Profile of the Content Standard for Digital Geospatial Metadata

Metadata\_Standard\_Version: FGDC-STD-001.1-1999

---

Generated by mp version 2.9.52 on Fri Mar 31 15:25:40 2023
