# Supplementary material for: With super SDMs (machine learning, open access big data, and the cloud) towards more holistic global squirrel hotspots and coldspots
Source: Sci Rep. 2024 Mar 3;14:5204. doi: 10.1038/s41598-024-55173-8 (PMC10909860; doi:10.1038/s41598-024-55173-8)
Supplement: Supplementary file 3 — Supplementary Information 3. [file 41598_2024_55173_MOESM3_ESM.docx]

| **Row Labels** | **Count of species** |
| --- | --- |
| *Aeretes melanopterus* | 2 |
| *Aeromys tephromelas* | 14 |
| *Aeromys thomasi* | 48 |
| *Ammospermophilus harrisii* | 1,435 |
| *Ammospermophilus insularis* | 12 |
| *Ammospermophilus interpres* | 240 |
| *Ammospermophilus jeffriesi* | 1 |
| *Ammospermophilus leucurus* | 4137 |
| *Ammospermophilus nelsoni* | 319 |
| *Arctomys nevadensis* | 1 |
| *Atlantoxerus getulus* | 1,350 |
| *Atlantoxerus rhodius* | 2 |
| *Belomys pearsonii* | 9 |
| *Blackia miocaenica* | 5 |
| *Blackia ulmensis* | 1 |
| *Callosciurus adamsi* | 8 |
| *Callosciurus baluensis* | 13 |
| *Callosciurus caniceps* | 279 |
| *Callosciurus erythraeus* | 2,373 |
| *Callosciurus finlaysonii* | 577 |
| *Callosciurus inornatus* | 14 |
| *Callosciurus melanogaster* | 2 |
| *Callosciurus nigrovittatus* | 68 |
| *Callosciurus notatus* | 1,652 |
| *Callosciurus orestes* | 62 |
| *Callosciurus phayrei* | 8 |
| *Callosciurus prevostii* | 272 |
| *Callosciurus pygerythrus* | 172 |
| *Callosciurus quinquestriatus* | 4 |
| *Callospermophilus lateralis* | 7,383 |
| *Callospermophilus madrensis* | 36 |
| *Callospermophilus saturatus* | 706 |
| *Cedromus savannae* | 2 |
| *Cedromus wardi* | 2 |
| *Citellus bensoni* | 4 |
| *Citellus cochisei* | 2 |
| *Citellus dotti* | 1 |
| *Citellus gidleyi* | 1 |
| *Citellus howelli* | 1 |
| *Citellus junturensis* | 2 |
| *Citellus matthewi* | 1 |
| *Citellus mcgheei* | 1 |
| *Citellus pattersoni* | 1 |
| *Citellus primitivus* | 1 |
| *Citellus quatalensis* | 2 |
| *Citellus rexroadensis* | 1 |
| *Citellus shotwelli* | 2 |
| *Citellus wilsoni* | 1 |
| *Comtia bernardi* | 1 |
| *Cryptopterus webbi* | 2 |
| *Cynomyoides vatis* | 1 |
| *Cynomys gunnisoni* | 915 |
| *Cynomys leucurus* | 549 |
| *Cynomys ludovicianus* | 4,714 |
| *Cynomys mexicanus* | 2,109 |
| *Cynomys niobrarius* | 5 |
| *Cynomys parvidens* | 189 |
| *Cynomys socialis* | 1 |
| *Cynomys spenceri* | 2 |
| *Dremomys everetti* | 51 |
| *Dremomys lokriah* | 63 |
| *Dremomys pernyi* | 115 |
| *Dremomys pyrrhomerus* | 13 |
| *Dremomys rufigenis* | 72 |
| *Eoglaucomys fimbriatus* | 13 |
| *Epixerus ebii* | 4 |
| *Eupetaurus cinereus* | 2 |
| *Eupetaurus nivamons* | 1 |
| *Eutamias ateles* | 6 |
| *Eutamias canipes* | 1 |
| *Eutamias malloryi* | 2 |
| *Exilisciurus concinnus* | 7 |
| *Exilisciurus exilis* | 131 |
| *Exilisciurus whiteheadi* | 54 |
| *Funambulus layardi* | 26 |
| *Funambulus palmarum* | 1,045 |
| *Funambulus pennantii* | 953 |
| *Funambulus sublineatus* | 112 |
| *Funambulus tristriatus* | 156 |
| *Funisciurus anerythrus* | 40 |
| *Funisciurus bayonii* | 3 |
| *Funisciurus carruthersi* | 35 |
| *Funisciurus congicus* | 74 |
| *Funisciurus isabella* | 7 |
| *Funisciurus lemniscatus* | 19 |
| *Funisciurus leucogenys* | 30 |
| *Funisciurus pyrropus* | 123 |
| *Funisciurus substriatus* | 38 |
| *Glaucomys sabrinus* | 1,267 |
| *Glaucomys volans* | 2,135 |
| *Glyphotes simus* | 1 |
| *Heliosciurus gambianus* | 435 |
| *Heliosciurus mutabilis* | 82 |
| *Heliosciurus punctatus* | 24 |
| *Heliosciurus rufobrachium* | 259 |
| *Heliosciurus ruwenzorii* | 24 |
| *Heliosciurus undulatus* | 60 |
| *Hesperopetes jamesi* | 1 |
| *Heteroxerus grivensis* | 2 |
| *Hylopetes alboniger* | 7 |
| *Hylopetes bartelsi* | 2 |
| *Hylopetes nigripes* | 7 |
| *Hylopetes phayrei* | 5 |
| *Hylopetes sagitta* | 3 |
| *Hylopetes sipora* | 1 |
| *Hylopetes spadiceus* | 24 |
| *Hylopetes winstoni* | 1 |
| *Hyosciurus heinrichi* | 2 |
| *Ictidomys mexicanus* | 352 |
| *Ictidomys parvidens* | 628 |
| *Ictidomys tridecemlineatus* | 2,640 |
| *Iomys horsfieldii* | 7 |
| *Kherem hsandgoliensis* | 1 |
| *Lariscus hosei* | 2 |
| *Lariscus insignis* | 48 |
| *Lariscus niobe* | 7 |
| *Lariscus obscurus* | 2 |
| *Marmota arizonae* | 1 |
| *Marmota baibacina* | 101 |
| *Marmota bobak* | 4,188 |
| *Marmota broweri* | 53 |
| *Marmota caligata* | 2,250 |
| *Marmota camtschatica* | 26 |
| *Marmota caudata* | 59 |
| *Marmota flaviventris* | 5,879 |
| *Marmota himalayana* | 174 |
| *Marmota kastschenkoi* | 85 |
| *Marmota marmota* | 10,325 |
| *Marmota menzbieri* | 1 |
| *Marmota monax* | 14,022 |
| *Marmota olympus* | 196 |
| *Marmota oregonensis* | 1 |
| *Marmota sawrockensis* | 3 |
| *Marmota sibirica* | 66 |
| *Marmota vancouverensis* | 55 |
| *Menetes berdmorei* | 104 |
| *Microsciurus alfari* | 52 |
| *Microsciurus flaviventer* | 65 |
| *Microsciurus mimulus* | 44 |
| *Microsciurus santanderensis* | 41 |
| *Miopetaurista albanensis* | 1 |
| *Miopetaurista dehmi* | 4 |
| *Miopetaurista thaleri* | 1 |
| *Miospermophilus wyomingensis* | 1 |
| *Myosciurus pumilio* | 8 |
| *Nannosciurus melanotis* | 22 |
| *Neotamias ruficaudus* | 41 |
| *Notocitellus adocetus* | 134 |
| *Notocitellus annulatus* | 160 |
| *Notosciurus granatensis* | 621 |
| *Nototamias quadratus* | 3 |
| *Otospermophilus argonautus* | 1 |
| *Otospermophilus atricapillus* | 59 |
| *Otospermophilus beecheyi* | 18,883 |
| *Otospermophilus variegatus* | 8,596 |
| *Paenemarmota barbouri* | 5 |
| *Palaearctomys bryanti* | 2 |
| *Palaeosciurus feignouxi* | 4 |
| *Palaeosciurus goti* | 5 |
| *Palaeosciurus sutteri* | 2 |
| *Paratamias tarassus* | 1 |
| *Paraxerus alexandri* | 17 |
| *Paraxerus boehmi* | 104 |
| *Paraxerus cepapi* | 1,210 |
| *Paraxerus cooperi* | 6 |
| *Paraxerus flavovittis* | 15 |
| *Paraxerus inuus* | 1 |
| *Paraxerus lucifer* | 21 |
| *Paraxerus ochraceus* | 223 |
| *Paraxerus palliatus* | 149 |
| *Paraxerus poensis* | 33 |
| *Paraxerus vexillarius* | 24 |
| *Paraxerus vincenti* | 3 |
| *Petaurista alborufus* | 32 |
| *Petaurista caniceps* | 8 |
| *Petaurista elegans* | 11 |
| *Petaurista hainana* | 1 |
| *Petaurista lena* | 78 |
| *Petaurista leucogenys* | 18 |
| *Petaurista magnificus* | 9 |
| *Petaurista mishmiensis* | 1 |
| *Petaurista nobilis* | 4 |
| *Petaurista petaurista* | 183 |
| *Petaurista philippensis* | 135 |
| *Petaurista xanthotis* | 3 |
| *Petaurista yunanensis* | 4 |
| *Petauristodon pattersoni* | 1 |
| *Petinomys crinitus* | 1 |
| *Petinomys fuscocapillus* | 1 |
| *Petinomys genibarbis* | 1 |
| *Petinomys hageni* | 5 |
| *Petinomys lugens* | 1 |
| *Petinomys mindanensis* | 4 |
| *Petinomys setosus* | 10 |
| *Petinomys vordermanni* | 3 |
| *Poliocitellus franklinii* | 620 |
| *Prosciurillus leucomus* | 16 |
| *Prosciurillus murinus* | 20 |
| *Prosciurillus rosenbergii* | 3 |
| *Prosciurillus topapuensis* | 1 |
| *Prosciurillus weberi* | 2 |
| *Protosciurus mengi* | 1 |
| *Protospermophilus kelloggi* | 3 |
| *Protoxerus aubinnii* | 27 |
| *Protoxerus stangeri* | 288 |
| *Pteromys momonga* | 4 |
| *Pteromys volans* | 5,959 |
| *Pteromyscus pulverulentus* | 8 |
| *Ratufa affinis* | 210 |
| *Ratufa bicolor* | 625 |
| *Ratufa indica* | 535 |
| *Ratufa macroura* | 323 |
| *Ratufa maelongensis* | 1 |
| *Rheithrosciurus macrotis* | 4 |
| *Rhinosciurus laticaudatus* | 18 |
| *Rubrisciurus rubriventer* | 2 |
| *Sciurillus pusillus* | 51 |
| *Sciuropterus minimus* | 1 |
| *Sciurotamias davidianus* | 42 |
| *Sciurus aberti* | 1,770 |
| *Sciurus aestuans* | 757 |
| *Sciurus alleni* | 507 |
| *Sciurus angusticeps* | 2 |
| *Sciurus annulatus* | 1 |
| *Sciurus anomalus* | 549 |
| *Sciurus arizonensis* | 772 |
| *Sciurus aureogaster* | 4,401 |
| *Sciurus ballovianus* | 3 |
| *Sciurus bredai* | 36 |
| *Sciurus carolinensis* | 161,481 |
| *Sciurus colliaei* | 515 |
| *Sciurus deppei* | 817 |
| *Sciurus flammifer* | 1 |
| *Sciurus gibberosus* | 1 |
| *Sciurus giganteus* | 1 |
| *Sciurus gilvigularis* | 5 |
| *Sciurus granatensis* | 2,421 |
| *Sciurus griseus* | 6,158 |
| *Sciurus ignitus* | 72 |
| *Sciurus igniventris* | 74 |
| *Sciurus lis* | 111 |
| *Sciurus malheurensis* | 1 |
| *Sciurus nayaritensis* | 386 |
| *Sciurus niger* | 48,402 |
| *Sciurus oculatus* | 205 |
| *Sciurus olsoni* | 1 |
| *Sciurus pucheranii* | 90 |
| *Sciurus pyrrhinus* | 10 |
| *Sciurus richmondi* | 9 |
| *Sciurus rufiventer* | 2 |
| *Sciurus sanborni* | 3 |
| *Sciurus spadiceus* | 146 |
| *Sciurus stramineus* | 376 |
| *Sciurus tephrus* | 1 |
| *Sciurus variegatoides* | 2,426 |
| *Sciurus vetustus* | 6 |
| *Sciurus vulgaris* | 200,696 |
| *Sciurus yucatanensis* | 816 |
| *Similisciurus maxwelli* | 2 |
| *Spermophilinus besanus* | 3 |
| *Spermophilinus giganteus* | 1 |
| *Spermophilopsis leptodactylus* | 20 |
| *Spermophilus alashanicus* | 21 |
| *Spermophilus boothi* | 1 |
| *Spermophilus brevicauda* | 5 |
| *Spermophilus citellus* | 3,373 |
| *Spermophilus cyanocittus* | 3 |
| *Spermophilus dauricus* | 45 |
| *Spermophilus erythrogenys* | 75 |
| *Spermophilus fulvus* | 74 |
| *Spermophilus jerae* | 1 |
| *Spermophilus major* | 182 |
| *Spermophilus musicus* | 49 |
| *Spermophilus orientalis* | 1 |
| *Spermophilus pallidicauda* | 18 |
| *Spermophilus pygmaeus* | 83 |
| *Spermophilus relictus* | 11 |
| *Spermophilus russelli* | 1 |
| *Spermophilus suslicus* | 209 |
| *Spermophilus taurensis* | 21 |
| *Spermophilus xanthoprymnus* | 136 |
| *Sundasciurus fraterculus* | 5 |
| *Sundasciurus hippurus* | 25 |
| *Sundasciurus hoogstraali* | 4 |
| *Sundasciurus jentinki* | 69 |
| *Sundasciurus juvencus* | 17 |
| *Sundasciurus lowii* | 47 |
| *Sundasciurus mindanensis* | 3 |
| *Sundasciurus moellendorffi* | 2 |
| *Sundasciurus philippinensis* | 26 |
| *Sundasciurus rabori* | 5 |
| *Sundasciurus samarensis* | 8 |
| *Sundasciurus steerii* | 14 |
| *Sundasciurus tahan* | 1 |
| *Sundasciurus tenuis* | 229 |
| *Syntheosciurus brochus* | 12 |
| *Tamias alpinus* | 147 |
| *Tamias amoenus* | 2,434 |
| *Tamias aristus* | 3 |
| *Tamias bulleri* | 71 |
| *Tamias canipes* | 96 |
| *Tamias cinereicollis* | 357 |
| *Tamias dorsalis* | 2,029 |
| *Tamias durangae* | 131 |
| *Tamias eviensis* | 1 |
| *Tamias merriami* | 1,345 |
| *Tamias minimus* | 4,791 |
| *Tamias obscurus* | 197 |
| *Tamias ochrogenys* | 126 |
| *Tamias palmeri* | 118 |
| *Tamias panamintinus* | 194 |
| *Tamias quadrimaculatus* | 216 |
| *Tamias quadrivittatus* | 1,022 |
| *Tamias ruficaudus* | 151 |
| *Tamias rufus* | 288 |
| *Tamias senex* | 416 |
| *Tamias sibiricus* | 11,888 |
| *Tamias siskiyou* | 131 |
| *Tamias sonomae* | 545 |
| *Tamias speciosus* | 913 |
| *Tamias striatus* | 27,790 |
| *Tamias townsendii* | 1,482 |
| *Tamias umbrinus* | 1,218 |
| *Tamiasciurus douglasii* | 5,588 |
| *Tamiasciurus hudsonicus* | 28,521 |
| *Tamiasciurus mearnsi* | 10 |
| *Tamiops maritimus* | 209 |
| *Tamiops mcclellandii* | 338 |
| *Tamiops rodolphii* | 58 |
| *Tamiops swinhoei* | 98 |
| *Trogopterus xanthipes* | 6 |
| *Urocitellus armatus* | 1,129 |
| *Urocitellus beldingi* | 1,147 |
| *Urocitellus brunneus* | 32 |
| *Urocitellus canus* | 92 |
| *Urocitellus columbianus* | 2,811 |
| *Urocitellus elegans* | 752 |
| *Urocitellus mollis* | 412 |
| *Urocitellus parryii* | 1,879 |
| *Urocitellus richardsonii* | 1,591 |
| *Urocitellus townsendii* | 124 |
| *Urocitellus undulatus* | 371 |
| *Urocitellus washingtoni* | 56 |
| *Xerospermophilus mohavensis* | 69 |
| *Xerospermophilus perotensis* | 49 |
| *Xerospermophilus spilosoma* | 1,519 |
| *Xerospermophilus tereticaudus* | 2,188 |
| *Xerus erythropus* | 620 |
| *Xerus inauris* | 241 |
| *Xerus princeps* | 38 |
| *Xerus rutilus* | 276 |
| **Grand Total** | **665,529** |
