# Supplementary material for: With super SDMs (machine learning, open access big data, and the cloud) towards more holistic global squirrel hotspots and coldspots
Source: Sci Rep. 2024 Mar 3;14:5204. doi: 10.1038/s41598-024-55173-8 (PMC10909860; doi:10.1038/s41598-024-55173-8)
Supplement: Supplementary file 4 — Supplementary Information 4. [file 41598_2024_55173_MOESM4_ESM.docx]

| **Predictor name** | **Source** | **Explanation** | **Citation** |
| --- | --- | --- | --- |
| BIO1_2_5min - BIO19_2_5min; tmin1 - tmin12; tmax1 - tmax12; tavg1 - tavg12; srad1 - srad12; prec1 - prec12; Wcaltitude | Worldclim (<https://www.worldclim.org/data/worldclim21.html>) | These datasets represent most of the climate data utilized for the SDM. | [1] |
| FAOCC | FAO Geonetwork (<http://www.fao.org/geonetwork/>) | This predictor represents the global climate classes. |  |
| LC12asc2 ; VE4 | Geospatial Information Authority of Japan (<https://www.gsi.go.jp/kankyochiri/gm_global_e.html>) | These predictors represent the global land cover (LC12asc2), and the global vegetation cover (VE4). |  |
| GlobalRiversProxy2 | A Simple Global River Bank full Width & Depth Database (<http://gaia.geosci.unc.edu/rivers/>) | This predictor represents all global mid-size and large rivers. | [2] |
| GlobalBigRivers11 | Global major rivers (<https://www.arcgis.com/home/item.html?id=44e8358cf83a4b43bc863646cd695945>) | This predictor represents all global large rivers. |  |
| GlobalCities2 | Global Cities (<https://hub.arcgis.com/datasets/6996f03a1b364dbab4008d99380370ed_0?geometry=-65.394%2C25.931%2C73.737%2C49.818>) | This predictor represents all global cities. |  |
| GlobalLakes2 | Global Lakes and Wetlands Database (GLWD) (<http://www.fao.org/land-water/land/land-governance/land-resources-planning-toolbox/category/details/es/c/1043160/>) | This predictor represents all global lakes and wetlands. |  |
| GlobalSnowCoverMonthJan2021_7; FFJan2020_3; FFFeb2020_3; FFMar2020_3; FFMay2020_3,FFJun2020_3; FFJul2020_3; FFAug2020_3; FFSep2020_3; FFOct2020_3; FFNov2020_3; FFJan2021_3 | Global Snow Cover and Forest fires (https://neo.sci.gsfc.nasa.gov/view.php?datasetId=MOD10C1_M_SNOW and <https://neo.sci.gsfc.nasa.gov/view.php?datasetId=MOD14A1_M_FIRE&year=2020>) | These predictors mainly represent the Global snow cover in the month of January, and the forest fire information for nearly all months of 2020 with exception of April and December as these months were not available. |  |
| WorldSoil2 | Global Soil characteristics map (<https://webarchive.iiasa.ac.at/Research/LUC/External-World-soil-database/HTML/HWSD_Data.html?sb=4>) | This predictor represents all global soil types and its characteristics. |  |
| WorldProtectedAreasMerged4 | Global Protected areas. (<https://www.protectedplanet.net/en/search-areas?geo_type=region&filters%5Bis_type%5D%5B%5D=terrestrial>) | This predictor represents all global protected areas merged into one shapefile. | [3] |
| WorldMammaldensity4; WorldRodentDensity3; WorldThreatenedMammalDensity3; GlobalBirdDensity2 | Global Mammal density. Proximity maps for the world mammal density, world rodent density, world threatened mammal density (<https://biodiversitymapping.org/index.php/mammals/>) | These predictors mainly represent global biodiversity densities. In detail, they contain the world mammal density, world rodent density, world bird density, and the world's threatened mammal density. | [4, 5] |
| GlobalRoadsProxy2 | Global Roads - Socioeconomic data and applications center (SEDAC) - Data center in NASA's Earth Observatory System Data and Information System (EOSDIS) (<https://sedac.ciesin.columbia.edu/data/set/groads-global-roads-open-access-v1/data-download>) | This predictor represents the global proximity to all world's roads. Minor roads may not be included. |  |
| HII1 | Human Influence Index (HII). (<https://sedac.ciesin.columbia.edu/data/set/wildareas-v2-human-influence-index-geographic/data-download>) | This predictor represents the global Human Influence index |  |
| WorldSlope1 | Slope. (<https://scholarworks.alaska.edu/handle/11122/7151>) | This predictor represents the global terrestrial and aquatic slope. | [6] |
| World_MAX_RH_JAN - World_MAX_RH_DEC; World_MIN_RH_JAN - World_MIN_RH_DEC | Global Monthly Relative Humidity. (<http://palebludata.com/?q=data>) | This predictor set represents the global maximum and minimum relative humidity for the months January to December of the year 2020. | [7] |

**Appendix 4 References:**

Fick, S. E., & Hijmans, R. J. WorldClim 2: new 1‐km spatial resolution climate surfaces for global land areas. *International journal of climatology*, **37**(12), 4302-4315 (2017).

Andreadis, K. M., Schumann, G. J. P., & Pavelsky, T. A simple global river bankfull width and depth database. *Water Resources Research*, **49**(10), 7164-7168 (2013).

UNEP-WCMC and IUCN. Protected Planet: The World Database on Protected Areas (WDPA) [Online], September 2020, Cambridge, UK: UNEP-WCMC and IUCN. Available at: [www.protectedplanet.net](http://www.protectedplanet.net). (2020)

Jenkins, C. N., Pimm, S. L., & Joppa, L. N. Global patterns of terrestrial vertebrate diversity and conservation. *Proceedings of the National Academy of Sciences*, **110**(28), E2602-E2610 (2013).

Pimm, S. L. et al. The biodiversity of species and their rates of extinction, distribution, and protection. *Science*, **344**(6187), 1246752 (2014).

Sriram, S., & Huettmann, F. A Global Model of Predicted Peregrine Falcon (Falco peregrinus) Distribution with Open Source GIS Code and 104 Open Access Layers for use by the global public. *Earth System Science Data Discussions*, 1-39 (unpublished).

Jones, P., & Wint, W. *Data set produced by Waen Associates for Environmental Research Group Oxford, Limited, funded by the International Research Consortium on Dengue Risk Assessment, Management and Surveillance (IDAMS),* European Union’s Seventh Framework Programme for research, technological development and demonstration under grant agreement no 281803 (2015).
