# Supplementary material for: With super SDMs (machine learning, open access big data, and the cloud) towards more holistic global squirrel hotspots and coldspots
Source: Sci Rep. 2024 Mar 3;14:5204. doi: 10.1038/s41598-024-55173-8 (PMC10909860; doi:10.1038/s41598-024-55173-8)
Supplement: Supplementary file 7 — Supplementary Legends. [file 41598_2024_55173_MOESM7_ESM.docx]

**List of Appendices in Supplemental Materials**

Appendix 1: R script to obtain GBIF occurrence points utilizing the RGBIF package.

Appendix 2: ISO-compliant metadata

Appendix 3: Squirrel species list with occurrence record counts

Appendix 4: Environmental predictors description (Reproduced Table 3.2 from Steiner and Huettmann in 2023)

Appendix 5: Documented R script from the Maxent Cloud computing run

Appendix 6: TIFF raster file of the produced global SDM
